# Mapping the multigenomic human system: structural asymmetry and interface gaps in host–exogenous biological interactions

**DOI:** 10.3389/fmicb.2026.1834677

**Published:** 2026-06-18

**Authors:** João Francisco Pollo Gaspary, Luis Felipe Dias Lopes, Fernanda Peron Gaspary, Eduarda Grando Lopes, Alfred Lee Edgar, Eduardo Poletti Camara, Antonio Geraldo Camara

**Affiliations:** 1 Institute AuBento—Center for Education, Clinical Practice, and Research in Orthomolecular and Integrative Medicine, Santa Maria, Brazil; 2 Postgraduate Program in Administration and Accounting, Center for Social and Human Sciences, Federal University of Santa Maria, Santa Maria, Brazil; 3 Franciscan University, Santa Maria, Brazil; 4 Veterinary Medicine Course, Federal University of Santa Maria, Santa Maria, Brazil; 5 Research and Development Department, ElastroCrete, LLC, Veyo, UT, United States; 6 Institute Camara—Center for Clinical and Orthomolecular Practice, Ribeirão Preto, Brazil

**Keywords:** membrane-level signal integration, multigenomic human system, multigenomic systems, systems microbiology, systems physiology

## Abstract

**Background:**

Host–microbiome research has expanded rapidly over the past two decades, generating extensive evidence linking microbial communities to immune regulation, metabolism, epithelial barrier integrity, and neuroendocrine signaling. Despite this progress, the organizational architecture through which exogenous biological signals become integrated into human physiological regulation remains comparatively under-synthesized. In particular, the regulatory interfaces connecting ecological microbial interaction with cellular and systemic physiological responses remain insufficiently integrated within the current literature.

**Objective:**

This study aimed to perform a structured synthesis of host–exogenous biological interaction in order to examine how evidence is distributed across distinct levels of biological integration and to evaluate whether the literature supports a coherent multigenomic interpretative framework for human physiological organization.

**Methods:**

A prospectively registered systematic synthesis was conducted using a Work Breakdown Structure (WBS)-based analytical architecture. Literature searches were organized into three predefined integration layers: functional physiological coupling, regulatory-interface mediation, and explicit genetic-level interaction. Following structured screening and architectural refinement, 168 studies were retained for cross-domain synthesis. Evidence was analyzed through sequential stages of structural mapping, cross-domain convergence analysis, and structural plausibility assessment.

**Results:**

The synthesis revealed a pronounced asymmetry within the evidentiary landscape. Functional host–microbe coupling is extensively consolidated across immune, metabolic, barrier, and neuroendocrine domains. In contrast, regulatory interfaces—particularly membrane-associated signaling environments and microenvironment-dependent regulatory dynamics—remain comparatively under-integrated. Cross-domain analysis identified recurrent stabilization-related processes involving barrier remodeling, immune recalibration, metabolic reprogramming, neuroendocrine coupling, and ecological signal amplification. These mechanisms frequently converged at membrane-associated signaling platforms operating within physicochemical microenvironments capable of shaping cellular decision processes.

**Conclusion:**

These findings support a systems-level interpretation in which the human organism may be understood as a symbiotic multigenomic system characterized by continuous signal integration across interacting genomic sources. Membrane-associated signaling interfaces appear to function as important regulatory nodes where ecological signals, host physiological state, and microenvironmental constraints interact to shape long-term physiological organization. Reframing host–exogenous biological interaction within this multigenomic systems perspective may therefore provide a conceptual foundation for future research investigating how stabilized regulatory configurations emerge and persist across human physiological systems.

## 1 Introduction

Over the past two decades, the study of host–microbe interactions has undergone a profound expansion, transforming the understanding of human physiology from a purely host-centric paradigm to one that increasingly recognizes the organism as an ecological system shaped by microbial coexistence. Advances in sequencing technologies and systems biology have revealed that microbial communities influence a wide range of physiological processes, including immune regulation (McFall-Ngai et al., 2013; Wiertsema et al., 2021), metabolic homeostasis (Valdes et al., 2018; Fan and Pedersen, 2021), epithelial barrier integrity (Lozupone et al., 2012; Relman, 2012), and neuroendocrine signaling (Cryan et al., 2019; Ahmed et al., 2022). This growing body of literature has firmly established that host physiology cannot be fully understood without considering the dynamic contributions of resident and transient microbial ecosystems (Douglas, 2010; Qin et al., 2010; Human Microbiome Project Consortium, 2012; Sender et al., 2016).

Within this context, the concept of a multigenomic human system has gained increasing relevance. In the present study, the term multigenomic system is used operationally to describe the coexistence and interaction of multiple genomic sources shaping physiological regulation, without presupposing a specific evolutionary framework such as the hologenome hypothesis.

From this perspective, human biological organization may be interpreted as reflecting interactions among multiple genetic layers, including the nuclear genome and mitochondrial genome (Mitchell, 1961; Wallace, 2012; Covarrubias et al., 2021), together with diverse microbial genomes inhabiting distinct ecological niches within the body (Human Microbiome Project Consortium, 2012; Sender et al., 2016; Liang and Bushman, 2021). Rather than acting as fully independent biological entities, these components appear to participate in complex ecological and molecular interaction networks capable of influencing physiological regulation and adaptive responses to environmental pressures (Douglas, 2010; Noble, 2012). This ecological interpretation of human biology has progressively informed contemporary microbiome research, motivating systems-level perspectives in which host physiological regulation is increasingly examined through dynamic interactions between host-derived and microbial molecular signals operating across multiple biological scales.

Recent conceptual syntheses have further proposed that persistent physiological configurations may reflect stabilized adaptive states emerging within integrated microbiome–human physiological systems, reinforcing the relevance of examining host–exogenous interaction through broader systems-level organizational frameworks (Gaspary et al., 2026).

Despite the rapid accumulation of studies linking microbial communities to physiological outcomes, the evidentiary architecture of host–exogenous biological interaction remains unevenly organized. Much of the literature has converged on functional associations between microbial activity and host physiological domains such as immune signaling (Medzhitov, 2008; Wiertsema et al., 2021), metabolic regulation (Hotamisligil, 2017; Fan and Pedersen, 2021), and neuroendocrine communication (Cryan et al., 2019; Ahmed et al., 2022). These functional couplings are now supported by numerous experimental studies and systematic syntheses, indicating that microbial influence on host physiology has achieved substantial conceptual consolidation at organ-system and network levels (Valdes et al., 2018; Lozupone et al., 2012; Relman, 2012).

However, the mechanisms through which microbial signals become operationally integrated within host regulatory systems remain comparatively under-synthesized. In particular, the regulatory interfaces that mediate the translation of ecological microbial signals into host cellular responses—such as membrane-level signaling platforms (Simons and Ikonen, 1997; Brown and London, 2000; Simons and Gerl, 2010), redox-mediated signaling environments (Verdin, 2015; Wang, 2012; Abrescia et al., 2020), and microenvironmental regulatory constraints—have received far less integrative attention. Similarly, the explicit participation of exogenous nucleic acids in host regulatory processes, including viral RNA sensing (Kleinman et al., 2012; Watanabe et al., 2011), microbial small RNA’s (He et al., 2018), and horizontal gene transfer mechanisms (Anderson and Seifert, 2011; Megens et al., 2014), has emerged in the literature but remains fragmented across isolated mechanistic studies.

This uneven consolidation suggests that host–microbiome research has progressed asymmetrically: functional physiological associations are extensively characterized, whereas the structural organization of regulatory-interface and genetic interaction layers remains insufficiently integrated within current conceptual frameworks (Douglas, 2010; Noble, 2012; McFall-Ngai et al., 2013; Liang and Bushman, 2021). As a consequence, the field possesses a rich descriptive landscape of host–microbiome associations but lacks a coherent structural representation of how exogenous biological signals traverse ecological, cellular, and genomic interaction levels within the human system (Handley, 2016; Ahmed et al., 2022; Zhou et al., 2022).

Addressing this gap requires approaches capable of mapping the evidentiary architecture of host–exogenous biological interaction rather than merely cataloguing thematic associations. Structural mapping of the literature can reveal how evidence is distributed across levels of biological organization and identify domains where conceptual integration remains incomplete. Such mapping is particularly relevant in the context of multigenomic system perspectives, which aim to connect ecological microbial interactions with molecular and genomic regulatory mechanisms (Kitano, 2002; Alon, 2007; Kauffman, 1993; Noble, 2012).

The present study therefore conducted a prospectively registered systematic synthesis designed to map structured layers of host–exogenous biological interaction within the human physiological system. Rather than reviewing individual mechanisms of host–microbe interaction, the objective of this study is to map the structural organization of the evidentiary landscape supporting these interactions across multiple levels of biological integration.

By reorganizing the literature through this structural lens, the study aims to characterize the distribution of evidence across integration layers and identify emergent mechanistic patterns that may inform future integrative frameworks for host–microbiome interaction.

## 2 Methods

To examine how host–exogenous biological interactions are represented across different levels of biological organization, the present study implemented a structured conceptual synthesis organized through a Work Breakdown Structure (WBS)-informed analytical framework (Project Management Institute, 2019; Project Management Institute, 2021). Rather than introducing WBS as a methodological innovation, it was employed as a procedural and organizational architecture to structure the analytical workflow and maintain analytical distinction between evidence retrieval, structural mapping, cross-domain integration, and systems-level synthesis. The overall evidence acquisition strategy followed established principles for systematic evidence synthesis and reporting transparency (Hulley et al., 2007; Page et al., 2021).

This framework enabled progressive decomposition of the research problem into analytically distinct yet methodologically interconnected components, consistent with structured analytical decomposition approaches commonly used in complex systems research (Kitano, 2002; Alon, 2007). The research process was partitioned into predefined Work Packages (WP1–WP5), allowing explicit differentiation between evidence acquisition, mechanistic mapping, cross-domain convergence analysis, and integrative consolidation.

**Figure 1:**
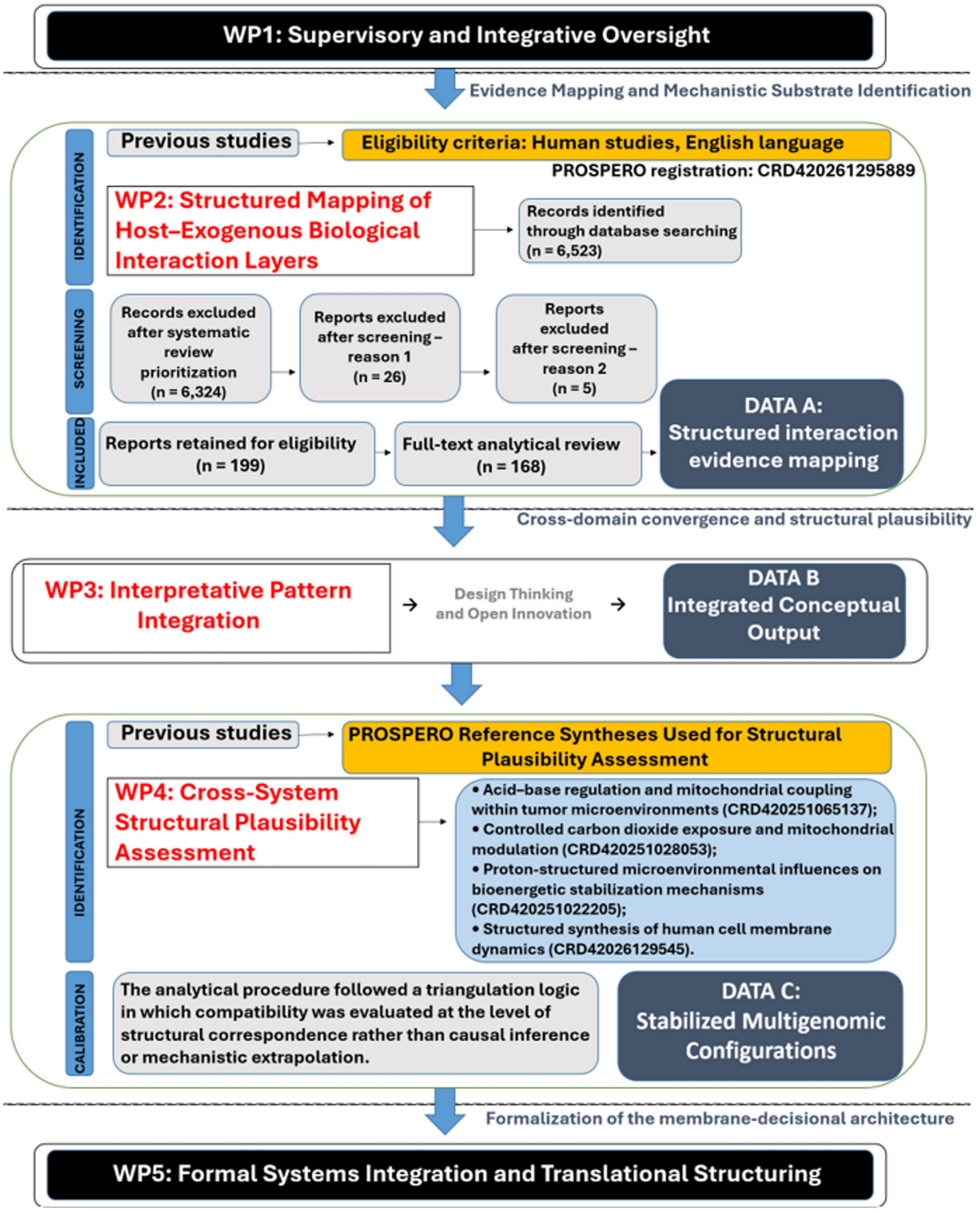
Structured analytical architecture of the study. This figure illustrates the structured analytical workflow implemented to investigate host–exogenous biological interaction across multiple levels of biological organization. The study design followed a Work Breakdown Structure (WBS) framework in which the research process was decomposed into sequential Work Packages (WP1–WP5), enabling explicit separation between evidence retrieval, structural mapping, cross-domain integration, and systems-level synthesis.

By structuring the analytical workflow in sequential stages, the design was intended to reduce interpretative overlap, maintain analytical traceability across integration layers, and enhance transparency in the progression from distributed empirical evidence toward systems-level interpretation. This staged analytical logic is consistent with systems-oriented approaches used to examine multilayer biological organization (Kitano, 2002; Noble, 2012). The resulting analytical architecture is illustrated in Figure 1.

### 2.1 WP1—supervisory and integrative oversight

WP1 functioned as a supervisory and integrative layer designed to preserve conceptual coherence and methodological alignment across all work packages. Rather than generating independent datasets, this stage operated as a structural oversight mechanism intended to ensure that evidence acquisition, analytical decomposition, and integrative interpretation remained sequential and analytically distinguishable throughout the study.

Within this supervisory framework, a progressive search refinement logic was applied when appropriate, enabling structured narrowing of evidentiary scope while preserving architectural representativeness across analytical layers. This approach ensured that subsequent work packages remained grounded in the evidentiary architecture established during the initial search strategy.

To further reduce interpretative bias and maintain inferential transparency, structured cross-disciplinary consultation was incorporated following open innovation principles (Chesbrough, 2003). These interactions were not used to introduce external interpretations, but rather to challenge preliminary analytical assumptions and reinforce epistemic discipline during successive stages of evidence synthesis. Such cross-domain analytical validation approaches are consistent with structured decision-analysis frameworks (Belton and Stewart, 2002) frequently applied in complex research environments (Simon, 1977).

WP1 therefore functioned as a procedural stabilization layer within the WBS architecture, maintaining conceptual continuity and methodological traceability across the analytical stages implemented throughout the study.

### 2.2 WP2—structured mapping of host–exogenous biological interaction layers

WP2 consisted of a prospectively registered systematic synthesis (PROSPERO CRD420261295889) designed for structural evidence mapping across layers through which exogenous biological systems and genetic material interact with human physiological processes [Centre for Reviews and Dissemination (CRD), 2026]. Rather than treating host–microbe interactions as a homogeneous evidentiary field, the synthesis was organized *a priori* into three predefined analytical layers representing increasing levels of biological integration. The objective of this stage was not quantitative evidence aggregation, but structural interpretation of how evidence is distributed and conceptually organized across distinct biological integration domains.

#### 2.2.1 Evidence structure and reporting parameters

The systematic components of this study followed PRISMA-informed parameters (Page et al., 2021) adapted to the multilayer analytical architecture defined by the WBS framework (Project Management Institute, 2019; Project Management Institute, 2021). Eligibility criteria prioritized mechanistic or functional evidence demonstrating physiologically relevant host–exogenous interactions across immune, endocrine, neural, metabolic, and barrier-related domains. Purely taxonomic descriptions, technical microbiome reports lacking physiological integration, and studies restricted to acute lethality without adaptive context were excluded. Animal and *in vitro* studies were retained only when providing mechanistic insight with plausible translational relevance to human physiology (Hulley et al., 2007).

Literature searches were conducted in PubMed/MEDLINE, Embase, and Web of Science using structured descriptor clusters aligned with the predefined analytical layers. Selection followed a two-stage filtering process emphasizing mechanistic coherence and structural representativeness rather than publication type alone.

Data extraction focused on the biological interface involved, the class of exogenous genetic source, the host physiological systems affected, and features related to adaptive stabilization. Rather than aggregating effect sizes, the synthesis prioritized identification of recurrent cross-domain organizational patterns across integration layers. This approach was adopted because the objective of the present synthesis was not to estimate pooled quantitative effects, but to structurally interpret how different levels of host–exogenous interaction are represented within the retained literature.

Given the integrative and cross-domain nature of the study, formal quantitative risk-of-bias instruments designed for intervention trials were not applied. Instead, methodological rigor was assessed qualitatively through transparency of evidence selection, cross-domain consistency, biological plausibility, and acknowledgment of known methodological limitations such as low-biomass contamination in microbiome research (Salter et al., 2014; Eisenhofer et al., 2019).

Extraction and consolidation of higher-order constructs during WP2 and WP3 followed structured evidence-convergence criteria, including cross-domain recurrence, mechanistic distinctiveness, and compatibility with the predefined integration layers. Higher-order constructs were therefore interpretatively consolidated through cross-domain convergence analysis rather than through quantitative weighting procedures.

#### 2.2.2 Search strategy

To ensure representation of major exogenous biological systems interacting with human physiology, descriptor clusters included bacterial, fungal (mycobiome), viral (virome), and parasitic organisms where appropriate (Human Microbiome Project Consortium, 2012; Liang and Bushman, 2021). The search architecture was therefore organized into three predefined analytical layers.

Layer I (Functional Integration) included descriptors targeting established physiological domains in which host–microbe interactions are extensively documented, including immune regulation (Wiertsema et al., 2021), endocrine signaling (Fan and Pedersen, 2021), barrier function (Lozupone et al., 2012), neurochemical modulation (Cryan et al., 2019), and metabolic–redox processes (Abrescia et al., 2020).

Layer II (Regulatory Interface) comprised descriptors addressing mechanisms through which microbial signals are operationally translated into host regulatory responses, including membrane-level signaling (Simons and Ikonen, 1997; Simons and Gerl, 2010), redox modulation (Wang, 2012), psychoneuroendocrine integration (Cryan et al., 2019), and adaptive response conditioning (Netea et al., 2016; Netea et al., 2020).

Layer III (Explicit Genetic-Level Interaction) included descriptors targeting direct participation of exogenous nucleic acids in host regulatory processes, including microbial small RNAs (He et al., 2018), viral RNA sensing (Watanabe et al., 2011), and other forms of host–exogenous genomic interaction (Zhou et al., 2022).

This layered search architecture enabled systematic differentiation between domain-level physiological integration, regulatory-interface mediation, and explicit genetic interaction prior to evidence synthesis.

For the purposes of this structured mapping, the term microbiome was operationally defined to encompass bacterial, fungal (mycobiome), archaeal, and protozoan communities inhabiting human-associated ecological niches (Human Microbiome Project Consortium, 2012; Sender et al., 2016).

The primary objectives of WP2 were therefore twofold: (i) to assess the extent of consolidation of functional host–microbe interactions across physiological domains, and (ii) to evaluate the systems-level representation of explicit host–exogenous genetic interaction within the broader evidentiary landscape. The structured distribution of search descriptors and retention outcomes across the predefined analytical layers is summarized in Table 1.

**Table 1:** Structured descriptor distribution and retention across predefined integration layers.

Layer	Descriptor combination	Records identified	Selected for structural synthesis
I – Functional integration	microbiome AND “human physiology”	415	9
	microbiome AND “human host”	491	1
	microbiome AND “neuroendocrine”	394	7
	microbiome AND “immune regulation”	767	6
	microbiome AND “immune modulation”	519	1
	microbiome AND “metabolic regulation”	227	2
	microbiome AND “barrier function”	1981	12
	microbiome AND “mitochondrial function”	256	4
	mycobiome AND “human physiology”	2	2
	mycobiome AND “immune regulation”	10	10
Layer I total		5,062	54
II – Regulatory interface	microbiome AND “psychoneuroendocrine”	3	1
	microbiome AND “endocrine signaling”	17	10
	microbiome AND “redox regulation”	9	3
	microbiome AND “membrane signaling”	2	1
	parasite AND “human physiology”	142	0
	parasite AND “human host”	1,103	7
	virome AND “human host”	19	14
	virome AND “human physiology”	12	8
	(parasite OR microbiome OR virome) AND “adaptive response”	78	65
	mycobiome AND “redox regulation”	0	0
	“fungal metabolites” AND “immune signaling”	0	0
Layer II total		1,385	109
III – Explicit genetic-level interaction	(“exogenous DNA” OR “exogenous RNA” OR “viral RNA”) AND “human host”	74	34
	“microbial small RNAs” AND “human host”	1	1
	“fungal extracellular vesicles” AND “human host”	1	1
Layer III total		76	36
Overall total		6,523	199

The descriptor architecture was intentionally designed to prioritize cross-domain coverage across predefined integration domains rather than exhaustive thematic saturation of all possible microbiome-related signaling pathways or molecular mediators. Descriptor combinations were selected to represent broad categories of host–exogenous interaction recurrently identified across immune, metabolic, neuroendocrine, barrier-related, regulatory-interface, and explicit genetic interaction domains within contemporary microbiome and systems physiology literature.

To reduce excessive concentration within highly published subdomains, broad mechanistic descriptors were preferentially used instead of highly specific molecular mediators or pathway-restricted terminology. Consequently, the search strategy was designed to support comparative structural mapping across integration layers rather than comprehensive thematic retrieval of all individual signaling molecules or microbiome-associated pathways.

The resulting descriptor distribution was subsequently evaluated through cross-domain representativeness and proportional coverage across integration layers rather than through exhaustive synonym expansion or pooled quantitative retrieval optimization.

The distribution of descriptor results across layers also reflects the uneven consolidation of the literature across different exogenous biological systems. While bacterial host–microbiome interaction has been extensively investigated across multiple regulatory levels, fungal (mycobiome) research remains predominantly concentrated in functional and immunological domains, with comparatively limited representation in regulatory-interface mechanisms.

#### 2.2.3 Selection logic

Duplicate records across descriptor combinations were removed prior to structural consolidation. Retention counts therefore reflect unique studies meeting predefined eligibility criteria.

A substantial reduction from 6,523 identified records to 199 retained studies resulted primarily from prioritizing systematic reviews and meta-analyses as integrative evidence units whenever available (Hulley et al., 2007). When a systematic synthesis was identified within a descriptor cluster, primary studies addressing the same mechanistic construct were not retained individually, as their evidentiary content was considered structurally represented within the integrative synthesis.

This approach prevented redundancy across highly synthesized domains and ensured balanced representation across the predefined analytical layers.

The reduction also reflects the distinction between descriptive publication density and structurally relevant evidence. Although the search architecture intentionally captured the full evidentiary landscape, many records within high-density domains—particularly barrier function and immune regulation—represented overlapping syntheses or descriptive expansions of already consolidated constructs.

Consequently, WP2 did not aim for thematic exhaustiveness within each descriptor cluster, but for structural representativeness across integration layers. Retention therefore prioritized studies introducing distinct mechanistic constructs relevant to adaptive stabilization rather than studies reiterating established domain-level associations.

Where systematic syntheses were available, they were preferentially retained as integrative evidence units. In contrast, for descriptor combinations lacking formal synthesis—particularly within the Regulatory Interface and Explicit Genetic Interaction layers—high-quality mechanistic primary studies were retained to avoid structural under-representation of emergent domains.

Following Round 1 selection (*n* = 199), a second-stage structural refinement was conducted to optimize architectural representativeness and minimize redundancy across descriptor domains. This process reduced the consolidated dataset to 168 unique studies.

The refinement process involved exclusion of studies based on two primary criteria. First, title, abstract, and targeted full-text assessment identified studies that did not demonstrate sufficient mechanistic or structural compatibility with the predefined analytical architecture. Second, studies presenting descriptive overlap without additional structural contribution beyond constructs already represented within the retained corpus were excluded. Throughout this stage, proportional coverage across the three analytical layers was preserved to reduce under-representation of domains characterized by lower publication density but higher mechanistic specificity.

**Figure 2:**
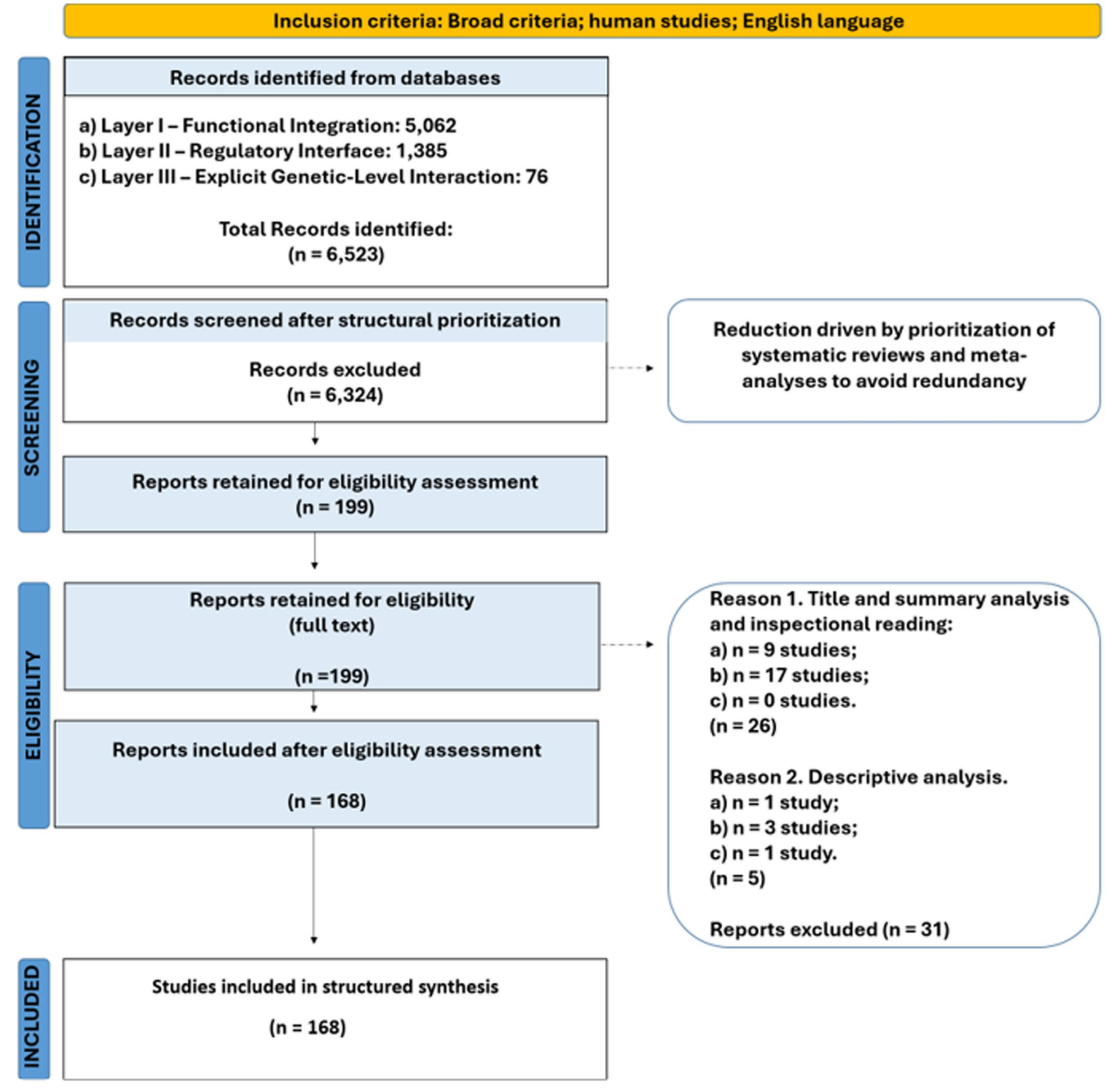
WP2 PRISMA-informed flow diagram of evidence selection and structural refinement across analytical integration layers. This figure illustrates the evidence selection and structural refinement workflow implemented during WP2. Records identified across the three predefined analytical integration layers underwent structural prioritization, eligibility assessment, and refinement based on mechanistic compatibility and reduction of descriptive redundancy. The process was designed to preserve proportional representativeness across integration layers while reducing overlap among highly synthesized domains.

This refinement process was intended to reduce redundancy while maintaining structural diversity and interpretative balance across integration layers rather than to perform additional evidentiary filtration. A detailed PRISMA-style representation of the selection and refinement process is provided in Figure 2. The resulting corpus of 168 studies constituted the structured evidentiary substrate for subsequent analytical stages, particularly the cross-domain synthesis conducted in WP3 and the structural plausibility assessment performed in WP4.

### 2.3 WP3—integrative pattern emergence

WP3 constituted a structured integrative synthesis designed to consolidate the mechanistic constructs identified during the structured mapping conducted in WP2. Rather than introducing new empirical datasets, this stage functioned as a convergence layer in which previously extracted constructs were examined through a formal cross-domain analytical framework.

The integrative process followed an iterative evidence-convergence logic consistent with systems-level analytical approaches used in complex biological integration (Kitano, 2002; Alon, 2007). Mechanistic variables derived from WP2 were initially decomposed into operational descriptors without hierarchical assumptions, allowing analytical expansion across domains prior to convergence. These descriptors included immune setpoint modulation (Medzhitov, 2008; Netea et al., 2016), barrier gating dynamics (Lozupone et al., 2012; Relman, 2012), redox–metabolic conditioning (Verdin, 2015; Covarrubias et al., 2021), membrane microdomain recruitment patterns (Brown and London, 2000; Simons and Gerl, 2010), nucleic-acid routing competence (He et al., 2018; Zhou et al., 2022), and lipid remodeling variables (Abrescia et al., 2020; Bibby et al., 2020).

The analytical workflow followed a structured divergence–convergence process characteristic of design thinking approaches (Brown, 2008) and systems-oriented decision and analytical frameworks (Simon, 1977; Belton and Stewart, 2002). During the divergence phase, mechanistic descriptors were examined independently across physiological contexts to reduce premature consolidation. Subsequently, during the convergence phase, descriptors were evaluated for cross-domain correspondence and structural compatibility.

Structured cross-domain alignment was then performed to determine whether independently derived constructs demonstrated coherent structural relationships across host physiological systems. Evidence convergence was accepted only when constructs exhibited mechanistic compatibility within human cellular physiology and remained confined to the empirical scope established in WP2.

To preserve inferential discipline, convergence was retained only when supporting evidence was present across at least two independent analytical domains and demonstrated compatibility with experimentally grounded biological mechanisms (Noble, 2012; Zhou et al., 2022). Constructs that could not be supported by cross-domain recurrence were not retained in the integrative synthesis.

The resulting integrative architecture therefore reflects recurrent cross-domain correspondences identified across the retained literature and interpreted within a systems-level analytical framework rather than quantitative evidence aggregation or externally imposed theoretical deduction.

### 2.4 WP4 – cross-system structural plausibility assessment

WP4 was designed as a cross-system structural plausibility assessment stage. Its purpose was to examine whether the structural correspondences identified through the cross-domain integration performed in WP3 remained internally coherent when evaluated against well-characterized physiological phenomena involving durable microenvironment-dependent stabilization.

This stage did not seek to establish generalizability, quantify effect sizes, or introduce new empirical evidence. Instead, it functioned as a bounded consistency evaluation, assessing whether the integrative constructs derived from WP2 and WP3 remained structurally compatible with recognized mechanisms of long-term physiological reconfiguration arising from physicochemical and microenvironmental constraints (Noble, 2012; Sterling, 2012).

The assessment focused on stabilization-relevant variables that plausibly influence threshold behavior and state persistence within biological systems. These included acid–base modulation (Mitchell, 1961; Rich and Maréchal, 2010) proton dynamics (Wang, 2012), redox tone (Verdin, 2015; Covarrubias et al., 2021) membrane-level integration (Simons and Gerl, 2010; Shi et al., 2018) and mitochondrial coupling efficiency (Wallace, 2012; Shi et al., 2018). These variables represent widely documented physicochemical determinants capable of shaping regulatory responsiveness within cellular environments.

Methodologically, WP4 drew upon previously registered systematic reviews addressing microenvironment-dependent regulation and bioenergetic stabilization phenomena, including PROSPERO registrations CRD420251065137 (acid–base regulation and mitochondrial coupling in the tumor microenvironment), CRD420251028053 (controlled carbon dioxide exposure and mitochondrial modulation), CRD42026129545 (structured synthesis of human cell membrane dynamics), and CRD420251022205 (proton-structured microenvironmental modulation and bioenergetic stabilization mechanisms). These registrations were used strictly as structured reference frameworks to guide cross-domain comparison rather than as extensions of the evidentiary base established in WP2 and WP3. Their function was to provide calibration anchors during structural alignment while maintaining inferential discipline and minimizing interpretative drift [Centre for Reviews and Dissemination (CRD), 2026].

The analytical procedure followed a triangulation logic in which compatibility was evaluated at the level of structural correspondence rather than causal extrapolation, consistent with heuristic reasoning approaches commonly applied in complex systems (Simon, 1977; Gigerenzer and Gaissmaier, 2011). Constructs derived from earlier work packages were examined in relation to independently documented stabilization phenomena to determine whether the proposed structural relationships remained physiologically plausible across distinct biological contexts.

No additional mechanisms were introduced during this stage, and no claims were inferred beyond the empirical boundaries defined by the preceding work packages. WP4 therefore functioned as a structural calibration layer, verifying coherence under physicochemical microenvironmental constraints prior to the formal systems consolidation performed in WP5.

### 2.5 WP5—formal systems integration and translational structuring

WP5 integrated the structured evidence base generated in WP2 and WP3 with the cross-system structural plausibility calibration conducted in WP4, consolidating the preceding analytical stages into a formalized systems-level interpretative architecture. No new empirical datasets were introduced at this stage. Instead, WP5 synthesized previously examined constructs into a coherent multiscale framework while remaining confined to the analytical boundaries established throughout the preceding work packages (Kitano, 2002; Alon, 2007; Noble, 2012).

The structural domains formalized during this stage were not defined *a priori*, but were interpretatively formalized through progressive abstraction of recurrent cross-domain stabilization variables identified during earlier analytical stages. These domains reflect clustered convergence patterns observed across membrane-level dynamics, bioenergetic modulation, and systemic stabilization features, as revealed through the integrative alignment of evidence mapped in WP2, consolidated through convergence analysis in WP3, and examined through structural plausibility assessment in WP4. Such pattern formalization is consistent with principles of self-organization and multiscale biological integration described in complex adaptive systems (Kauffman, 1993; Noble, 2012).

To ensure translational coherence and research tractability, the resulting architecture was evaluated using established (Doran, 1981) and FINER criteria (Hulley et al., 2007). This evaluation did not constitute empirical validation, but rather a structured appraisal of conceptual viability, methodological tractability, and translational relevance within contemporary biomedical research constraints.

WP5 therefore represents the formal consolidation layer of the WBS analytical architecture (Project Management Institute, 2019; Project Management Institute, 2021), transforming recurrent cross-domain structural correspondences into an operationally bounded systems-level interpretative framework while preserving explicit analytical discipline.

The resulting systems architecture also provided the interpretative foundation for the translational reflection developed in Section 4, where the implications of the structural patterns identified across WP2–WP5 are examined within the broader context of symbiotic multigenomic physiological organization (Douglas, 2010; McFall-Ngai et al., 2013).

## 3 Results

The analytical workflow implemented in this study produced a structured evidentiary corpus that enabled examination of host–exogenous biological integration across multiple levels of biological organization. Results are presented according to the sequential analytical stages defined in the WBS architecture.

First, the structured mapping performed in WP2 characterizes how evidence is distributed across predefined integration layers within the literature. Second, the cross-domain synthesis conducted in WP3 identifies recurrent stabilization-related patterns observed across otherwise heterogeneous physiological contexts, consistent with systems-level integration processes described in biological network analysis. Third, the structural plausibility calibration performed in WP4 examines whether these convergent patterns remain physiologically coherent when evaluated against physicochemical and microenvironmental stabilization processes documented in independent biological systems. Finally, the integrative consolidation conducted in WP5 organizes these observations into a systems-level interpretative framework describing potential multigenomic stabilization dynamics within host–exogenous biological interaction.

The interpretative patterns presented throughout the Results section should therefore be understood as components of a structured systems-level synthesis derived from cross-domain evidence convergence rather than as definitive causal models of biological regulation.

Together, these analytical stages allow the literature to be examined not only as a collection of isolated findings, but also as an evidentiary landscape in which recurrent organizational correspondences across host–exogenous interaction domains become interpretatively visible.

### 3.1 Evidence consolidation and structural distribution across integration layers

The WP2 analysis of the retained corpus revealed a pronounced asymmetry in the distribution of evidence across the predefined integration layers. Studies addressing functional host–microbe coupling were highly represented, particularly within domains involving immune modulation (Wiertsema et al., 2021; Netea et al., 2016), epithelial barrier dynamics (Fan and Pedersen, 2021; Lozupone et al., 2012), metabolic regulation (Stefanaki et al., 2017; Valdes et al., 2018), and neuroendocrine signaling (Mayer et al., 2014; Cryan et al., 2019). These domains frequently appeared in systematic reviews and meta-analyses, indicating a substantial degree of consolidation at the level of physiological system interaction.

In contrast, literature addressing regulatory interfaces through which microbial signals are translated into host cellular responses was considerably less integrated. Mechanistic studies involving membrane-level signaling environments (Shi et al., 2018) redox-mediated regulatory modulation (Abrescia et al., 2020) and microenvironment-dependent response conditioning (Nathan and Ding, 2010; Sterling, 2012) were present but rarely synthesized into unified explanatory frameworks.

The third analytical layer, involving explicit genetic interaction between host systems and exogenous nucleic acids, demonstrated the lowest degree of systematic consolidation. Evidence describing viral RNA sensing (Obermann et al., 2022), microbial small RNA activity (He et al., 2018), and host responses to exogenous nucleic acid material (Zhou et al., 2022) appeared primarily in isolated mechanistic studies rather than in integrative syntheses.

Taken together, these patterns indicate that while host–microbe physiological coupling is extensively characterized in the literature, the mechanistic interfaces through which exogenous biological signals acquire regulatory influence within human systems remain comparatively under-integrated.

### 3.2 Recurrent stabilization mechanisms across domains

Across the consolidated corpus, the literature did not organize into isolated thematic clusters only, but also revealed recurrent mechanistic patterns that appeared across multiple physiological contexts. These patterns were not restricted to a single organ system, microbial niche, or disease category. Instead, they recurred as stabilization-relevant processes through which host–exogenous biological interactions appeared to acquire persistence over time. Although the specific molecular mediators varied across studies, the higher-order organizational behaviors remained remarkably convergent.

One recurrent pattern involved the dynamic regulation of biological barriers. Evidence from gastrointestinal, epithelial, and inflammatory contexts consistently indicated that barrier integrity was not a static structural property but a modifiable interface capable of shifting under ecological and inflammatory pressure. Changes in epithelial permeability, tight junction organization, mucus composition, and interface selectivity repeatedly appeared as mechanisms through which host systems adjusted their exposure to exogenous biological signals (Angriman et al., 2014; Touchefeu et al., 2014; Williamson et al., 2020; Buitrago-Ruiz et al., 2026). These alterations were documented in contexts such as inflammatory bowel disease, short bowel syndrome, chronic colitis, and environmentally perturbed gut ecosystems (Touchefeu et al., 2014; Engelstad et al., 2018; Zhang et al., 2020; Zhang et al., 2026), suggesting that barrier remodeling functions as a stabilization mechanism capable of reshaping host–exogenous interaction thresholds rather than merely reflecting passive damage.

A second recurrent pattern concerned recalibration of immune setpoints. Across infectious, inflammatory, and symbiotic contexts, studies consistently described persistent shifts in innate–adaptive balance, changes in cytokine profiles, and altered tolerance or vigilance thresholds (Netea et al., 2016; Netea et al., 2020). These changes did not simply represent activation or suppression in a binary sense. Rather, the literature repeatedly suggested that immune behavior was being reweighted toward alternative stable response regimes. This was particularly evident in studies involving helminth exposure, fungal symbiosis, virome-associated immune modulation, and chronic inflammatory conditions (Croese et al., 2013; Haarder et al., 2017; Iliev and Cadwell, 2021; Vietzen et al., 2024), where long-term adjustment of immune responsiveness appeared more relevant than acute pathogen clearance alone. The recurrence of this pattern across heterogeneous biological settings indicates that immune recalibration is a central mechanism through which adaptive stabilization may be sustained within host–exogenous systems.

A third recurrent pattern involved metabolic and redox reprogramming. Numerous studies linked host–microbe interaction to shifts in energetic routing, mitochondrial function, lipid metabolism, and redox-sensitive signaling environments. Importantly, these findings did not remain confined to canonical metabolism studies. Similar reorganization of metabolic tone was observed across inflammatory, neuroendocrine, and ecological adaptation contexts, suggesting that metabolic modulation operates as a shared substrate through which physiological states can be stabilized. Changes involving short-chain fatty acids, bile acid signaling, tryptophan-derived metabolites, taurine-related pathways, mitochondrial coupling, and oxidative balance repeatedly emerged as mechanistic correlates of persistent host adaptation (Armand et al., 2022; Dumitrescu et al., 2022; Sinha et al., 2024). This convergence suggests that metabolic regulation is not merely a downstream consequence of host–microbe interaction, but one of the principal media through which long-term stabilization is enacted.

Neuroendocrine coupling also emerged as a recurrent cross-domain feature. Evidence from gut–brain, endocrine, psychoneuroimmunological, and stress-adaptation studies indicated that microbial and exogenous biological signals repeatedly intersected with host neuroendocrine organization. This occurred through modulation of vagal signaling, enteroendocrine communication, hypothalamic–pituitary–adrenal axis responsiveness, and neurochemical precursor availability (Cryan et al., 2019; Ahmed et al., 2022; Dicks, 2023). Importantly, these interactions were not limited to acute signaling events. Across the retained corpus, they frequently appeared as processes capable of biasing longer-term physiological weighting, particularly in contexts involving chronic stress, persistent inflammatory activation, altered microbial ecology, or neuropsychiatric symptom persistence (Westfall et al., 2017; Carbone et al., 2020; Barrio et al., 2022). The recurrence of this pattern across otherwise distinct domains suggests that neuroendocrine coupling functions as an important integrative route through which exogenous biological information may become embedded into host adaptive states.

Another recurrent feature involved ecological selection pressure. Multiple retained studies described host–exogenous interaction not as a static biological association, but as a context-dependent process shaped by environmental constraints such as hypoxia, altitude, pollution exposure, toxic load, nutrient competition, and physicochemical stress (Roslund et al., 2019; Sturgess and Montgomery, 2021; Vari et al., 2021). In these contexts, biological systems appeared to reorganize around selective pressures that favored certain host–microbe configurations over others. This was particularly visible in studies addressing environmental adaptation, chronic inflammation under ecological stress, and microbial restructuring under altered host conditions (Zhu et al., 2025; Yan et al., 2025). Rather than acting merely as background modifiers, these pressures appeared as active stabilizing influences shaping which physiological configurations persisted.

The literature also revealed recurrent evidence that microbial and viral signals can participate more directly in host regulatory processes than is usually acknowledged in broad physiological syntheses. Although this evidence remained less systematically integrated than functional host–microbe associations, retained studies repeatedly pointed toward interactions involving exogenous nucleic acid sensing, microbial small RNAs, extracellular vesicle-associated RNA, and host editing or restriction responses (He et al., 2018; Inoue et al., 2020; Zhou et al., 2022). Such findings remained mechanistically fragmented across the corpus, but their recurrence across independent studies suggests that explicit genomic interaction is not absent from the field; rather, it remains under-consolidated relative to functional physiological coupling.

Virome-associated modulation represented another recurring pattern that extended beyond conventional pathogen-centered interpretation. Several retained studies suggested that viral and phage-related ecological dynamics can influence immune equilibrium, microbial community structure, and host signaling conditions without necessarily manifesting as acute infection (Handley, 2016; Liang and Bushman, 2021; Tiamani et al., 2022). This was especially notable in studies involving anelloviruses, Caudoviricetes, and broader virome-associated ecological regulation (Gulyaeva et al., 2022; Vietzen et al., 2024). These findings support the interpretation that host–exogenous integration cannot be adequately described through bacterial association alone, as viral and phage-mediated network effects also contribute to stabilization of the biological environment in which host responses unfold.

Finally, a recurrent pattern of signal amplification through microbial metabolites emerged across diverse physiological contexts. Rather than acting as isolated biochemical outputs, microbial metabolites repeatedly appeared in the literature as systemic amplifiers capable of extending local ecological processes into broader host physiological domains. Short-chain fatty acids, bile acids, indole derivatives, and related signaling intermediates were repeatedly implicated in immune modulation, metabolic routing, epithelial interface regulation, and neuroendocrine communication (Valdes et al., 2018; Ahmed et al., 2022; Dimba et al., 2024). Their recurrence across distinct literatures suggests that metabolite-mediated amplification is one of the principal routes through which local host–exogenous interactions become translated into broader patterns of adaptive stabilization.

Taken together, these findings indicate that the retained corpus is structured not only by thematic diversity, but by recurrence of a limited number of mechanistic stabilization behaviors that reappear across apparently unrelated physiological domains. The importance of this observation lies in the fact that these mechanisms do not describe isolated associations. Rather, they reveal a shared organizational logic through which host–exogenous interactions may acquire persistence, coherence, and cross-system influence. This recurrent mechanistic convergence provided the basis for the cross-domain integrative analysis conducted in WP3.

### 3.3 Cross-domain evidence convergence

The integrative synthesis conducted in WP3 revealed that the stabilization mechanisms identified across the retained corpus rarely operated in isolation. Instead, the literature consistently described configurations in which multiple regulatory processes appeared simultaneously within the same physiological contexts. These recurrent co-occurrences suggested that host–exogenous biological integration is not mediated through single pathways but through coordinated interaction across several regulatory layers.

Barrier modulation, immune recalibration, metabolic routing, and neuroendocrine signaling frequently appeared as interconnected processes rather than independent biological events. For example, studies examining gastrointestinal dysbiosis often simultaneously described altered epithelial permeability, immune tolerance shifts, microbial metabolite signaling, and neurochemical modulation within the same physiological context (Mayer et al., 2014; Stefanaki et al., 2017; Cryan et al., 2019; Dicks, 2023). Similar convergence patterns appeared in studies addressing chronic inflammatory disorders, metabolic syndromes, and neuropsychiatric conditions (Medzhitov, 2008; Liu et al., 2021; Valencia et al., 2025; Banerjee et al., 2026).

This cross-domain alignment suggests that physiological stabilization in host–exogenous systems is rarely achieved through a single dominant mechanism. Rather, stabilization appears to emerge from coordinated adjustments occurring at multiple biological interfaces simultaneously. Barrier dynamics regulate the exposure of host tissues to microbial signals, immune thresholds recalibrate the interpretation of these signals, metabolic pathways adjust the energetic environment in which these interactions occur, and neuroendocrine circuits integrate systemic responses to these conditions (Valencia et al., 2025).

The recurrence of these coupled processes across otherwise distinct biological contexts indicates that host–exogenous interaction cannot be adequately interpreted through isolated functional pathways. Instead, the evidence suggests the presence of multi-interface stabilization dynamics in which ecological signals, cellular signaling platforms, and systemic regulatory circuits converge to maintain coherent physiological states (Gold and Shadlen, 2007; Noble, 2012).

Importantly, these convergence patterns did not imply uniform mechanistic sequences across studies. Different biological systems appeared to emphasize distinct combinations of regulatory processes depending on ecological context, microbial composition, host physiological condition, and environmental pressures. Nevertheless, the recurrence of similar stabilization behaviors across independent domains suggests that host–exogenous integration operates through a limited repertoire of regulatory configurations that reappear across diverse physiological settings (Lozupone et al., 2012; Relman, 2012; Sender et al., 2016; Rampelli et al., 2017; Yan et al., 2025).

These findings therefore support the interpretation that the literature documents not only a diversity of host–microbe associations but also a set of recurring structural behaviors through which host–exogenous biological interactions acquire persistence and cross-system influence. Identification of these convergence patterns provided the analytical basis for the structural plausibility assessment conducted in WP4.

### 3.4 Structural plausibility under physicochemical microenvironmental conditions

The structural plausibility assessment conducted in WP4 examined whether the convergence patterns identified through cross-domain synthesis remained coherent when considered in relation to well-characterized physicochemical stabilization processes documented in independent physiological systems.

Across multiple biological contexts, the literature consistently describes physiological states that remain stable not because a single regulatory pathway dominates, but because local physicochemical conditions constrain the range of viable biological responses. Variables such as pH gradients (Mitchell, 1961; Rich and Maréchal, 2010), redox balance (Nathan and Ding, 2010; Abrescia et al., 2020), oxygen availability (Wallace, 2012), proton dynamics (Verdin, 2015), and mitochondrial coupling efficiency (Verdin, 2015; Covarrubias et al., 2021) repeatedly appear in experimental and translational research as determinants of how cellular signaling platforms interpret and respond to environmental inputs.

When the convergence patterns identified in WP3 were examined within this broader physiological context, the stabilization mechanisms described in the retained corpus appeared compatible with known microenvironment-dependent regulatory constraints. For example, barrier permeability shifts (Schroeder et al., 2011; Iliev and Cadwell, 2021) and immune recalibration (Medzhitov, 2008; Netea et al., 2020) described in host–microbe interaction studies frequently occur in parallel with alterations in redox tone (Ding et al., 2013) and local metabolic conditions (Hotamisligil, 2017) that influence epithelial signaling thresholds. Similarly, metabolic reprogramming (Fan and Pedersen, 2021) and microbial metabolite signaling (Ahmed et al., 2022) identified across the corpus are consistent with well-documented roles of mitochondrial coupling efficiency (Rich and Maréchal, 2010) and proton gradients (Mitchell, 1961) in shaping cellular responsiveness to external signals.

In several physiological systems, stable functional configurations are known to emerge when microenvironmental parameters remain within constrained ranges that favor certain signaling pathways while limiting others. Tumor microenvironment studies (Wallace, 2012), chronic inflammatory tissue models (Liu et al., 2021), and metabolic adaptation research (Hotamisligil, 2017) all describe conditions in which alterations in acid–base balance (Wang, 2012), oxygen tension (Wallace, 2012), or redox dynamics (Abrescia et al., 2020) reorganize cellular behavior without requiring genetic mutation or structural damage. These examples demonstrate that durable physiological configurations can arise from persistent physicochemical conditions that shape signaling environments over time.

The patterns identified in the present synthesis align with these observations. The stabilization mechanisms observed across host–exogenous interaction studies frequently appear in biological contexts where microenvironmental constraints are capable of biasing cellular decision processes toward specific regulatory outcomes. Importantly, these findings do not imply that physicochemical conditions alone determine physiological states. Rather, they suggest that host–exogenous interaction occurs within a physicochemical field that constrains the range of biologically sustainable configurations.

Within this interpretative frame, host–exogenous biological integration can be understood as unfolding within environments where ecological signals, cellular signaling platforms, and physicochemical constraints interact simultaneously. The structural convergence identified in WP3 therefore remains physiologically plausible when examined against the broader body of evidence describing microenvironment-dependent stabilization phenomena across human biological systems.

These observations support the interpretation that the recurrent stabilization mechanisms identified in the retained corpus are compatible with established principles of microenvironment-dependent regulation, thereby providing a consistent physiological context for the systems-level integration performed in WP5.

### 3.5 Systems-level integration of host–exogenous biological interaction

The final analytical stage integrated the evidence distribution identified in WP2, the cross-domain convergence patterns revealed in WP3, and the structural plausibility assessment conducted in WP4 in order to examine whether the retained corpus collectively described a coherent systems-level organization of host–exogenous biological interaction.

When examined together, the evidence did not suggest isolated biological associations but rather a structured pattern in which host physiological regulation appeared repeatedly intertwined with signals originating from exogenous biological systems. Across the literature, microbial populations, viral elements, and other exogenous genomic components were consistently associated with modulation of immune thresholds (Eisenhofer et al., 2019; Wiertsema et al., 2021), barrier permeability (Lozupone et al., 2012; Angriman et al., 2014), metabolic routing (Fan and Pedersen, 2021; Valencia et al., 2025) and neuroendocrine signaling (Cryan et al., 2019; Dicks, 2023). These processes did not appear as independent pathways but as interconnected regulatory interfaces through which ecological signals could influence host physiological organization.

This integration became particularly visible when stabilization mechanisms identified across domains were considered simultaneously. Barrier remodeling influenced exposure of host tissues to microbial signals; immune recalibration modified interpretation of these signals; metabolic pathways reshaped the energetic environment in which host–microbe interactions unfolded; and neuroendocrine circuits integrated these conditions into systemic physiological responses. The recurrent co-occurrence of these processes across diverse physiological contexts suggested that host–exogenous interaction operates through coordinated multi-interface regulation rather than through single mechanistic pathways.

At the same time, the literature repeatedly indicated that exogenous biological influence is not limited to extracellular signaling. Evidence describing viral RNA sensing (Obermann et al., 2022), microbial small RNA activity (He et al., 2018), and other nucleic acid–mediated interactions (Zhou et al., 2022) suggested that genomic material originating outside the host may also participate directly in regulatory processes affecting cellular behavior. Although this layer of interaction remained less consolidated than functional host–microbe coupling, its recurrence across independent studies indicated that genetic interaction represents an additional dimension of host–exogenous integration that remains incompletely synthesized within current conceptual frameworks.

Taken together, these observations suggest that the human organism is best interpreted not as a purely host-driven biological system transiently influenced by external agents, but as a symbiotic configuration in which host cellular processes operate within a broader ecological and genomic environment. Within this configuration, physiological regulation emerges from continuous interaction between host genetic programs and signals derived from exogenous genomic populations inhabiting shared biological niches.

The integration performed in WP5 therefore indicates that the retained corpus collectively describes a multigenomic biological system in which host physiology and exogenous biological activity interact across ecological, cellular, and genomic layers. Rather than representing isolated perturbations, these interactions appear capable of stabilizing coherent physiological configurations that persist across time and biological context (Relman, 2012; Sender et al., 2016; Valencia et al., 2025).

This systems-level organization provides the empirical basis for the interpretative considerations developed in the following section, where the implications of these structural patterns are examined in relation to long-term physiological stabilization and translational research perspectives.

**Figure 3:**
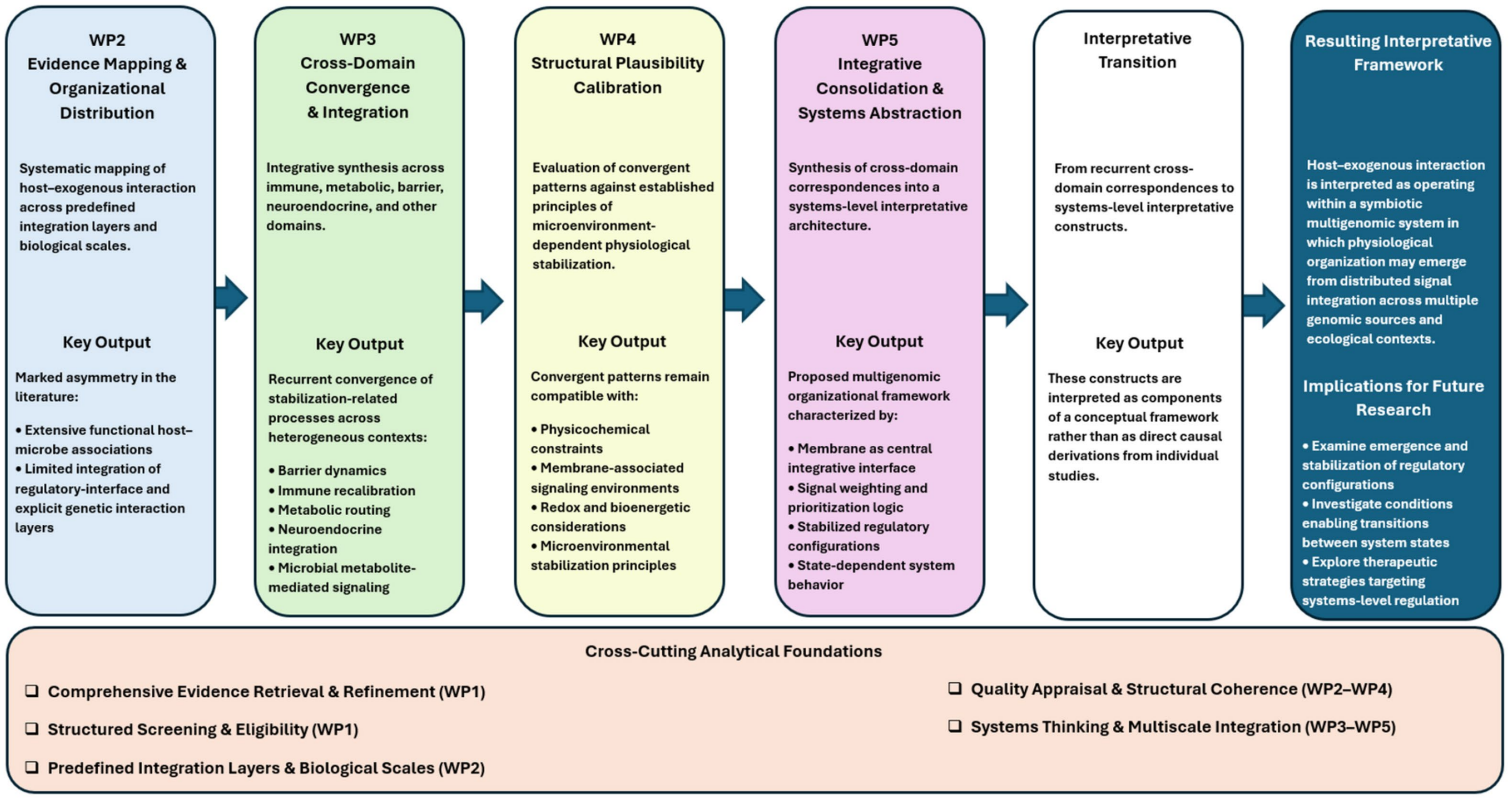
Interpretative progression from structured evidence synthesis to multigenomic systems-level framework. This figure illustrates the interpretative progression through which recurrent cross-domain organizational correspondences identified across WP2–WP4 were progressively consolidated into a systems-level conceptual framework in WP5. WP2 maps asymmetries and organizational distribution patterns across predefined host–exogenous interaction layers. WP3 integrates recurrent stabilization-related correspondences observed across heterogeneous physiological domains, while WP4 evaluates whether these convergent patterns remain compatible with independently established principles of microenvironment-dependent physiological stabilization.

The interpretative progression through which recurrent cross-domain organizational correspondences identified across WP2–WP4 were consolidated into the systems-level framework proposed in WP5 is summarized in Figure 3.

WP5 consolidates these interpretative correspondences into a systems-level organizational framework emphasizing membrane-associated signal integration, stabilization dynamics, and multigenomic physiological organization. The resulting constructs should therefore be understood as structured interpretative abstractions derived from cross-domain evidence convergence rather than as direct causal derivations from isolated studies.

## 4 Translational reflection on structural findings

The following interpretative reflection derives from the structured cross-domain synthesis achieved across WP2–WP5 and examines the potential implications of the identified organizational correspondences for understanding host–exogenous interaction within symbiotic multigenomic systems.

In methodological terms, the present section develops through two sequential interpretative layers: first, a bounded plausibility reflection aligned with WP4, and second, a systems-level conceptual consolidation corresponding to WP5. The interpretations developed throughout this section should therefore be understood as components of a structured systems-oriented framework derived from cross-domain evidence convergence rather than as definitive causal explanations of biological regulation.

The recurrent organizational patterns identified across the preceding analytical stages invite reconsideration of how host–exogenous biological interaction may be interpreted within biomedical research. The evidence synthesized in WP2 revealed a pronounced asymmetry in the consolidation of the literature, with functional host–microbe coupling extensively characterized across physiological domains, while regulatory-interface and explicit genetic interaction layers remain comparatively under-integrated. Subsequent cross-domain convergence analysis in WP3 indicated that stabilization-related processes recurrently co-occur across barrier dynamics, immune recalibration, metabolic routing, and neuroendocrine integration. Finally, the plausibility assessment conducted in WP4 suggested that these convergent patterns remain compatible with established principles of microenvironment-dependent physiological stabilization.

Taken together, these observations support the interpretative possibility that host–exogenous biological interaction may not be fully captured by models treating microbial or environmental factors as isolated modulators of host physiology. Instead, the retained literature recurrently describes configurations in which host regulatory processes operate within ecological and physicochemical environments capable of reshaping signaling thresholds, metabolic conditions, and systems-level responsiveness. Within such contexts, physiological organization may be more appropriately interpreted as emerging from distributed interactions across multiple regulatory interfaces rather than from host genetic programs alone (Handley, 2016; Hotamisligil, 2017; Valdes et al., 2018; Liang and Bushman, 2021).

This interpretative perspective has important implications for how chronic physiological states may be conceptualized. If host–exogenous interaction unfolds within integrated ecological and genomic environments, persistent physiological configurations may reflect stabilized systems-level organization rather than transient dysregulation alone. In this sense, long-term pathological conditions may be interpreted not exclusively as failures of regulatory mechanisms, but potentially as stabilized adaptive regimes emerging under altered ecological constraints (Kauffman, 1993; Noble, 2012; Relman, 2012; Sterling, 2012).

The translational relevance of this perspective lies in its potential capacity to reinterpret a wide range of biomedical observations that remain difficult to reconcile within strictly host-centered models. Chronic inflammatory persistence, recurrent infection despite pathogen clearance, resistance to targeted interventions, and heterogeneous responses to nutritional or metabolic correction may all reflect the presence of stabilized physiological configurations in which host regulatory systems operate under altered ecological weighting (Franceschi et al., 2017).

Rather than invalidating established mechanistic knowledge, the present framework proposes that many well-documented molecular and cellular processes may represent components of broader systems-level organizational configurations. Understanding how such configurations stabilize—and under what conditions they may be renegotiated—therefore emerges as a potentially important translational question for future biomedical research (Kitano, 2002; Alon, 2007; Gold and Shadlen, 2007).

### 4.1 From eradication paradigms to symbiotic system organization

A recurrent assumption underlying many therapeutic strategies in modern medicine is that pathological states can be resolved through elimination of the biological agents associated with them. This logic, often implicit within antimicrobial, anti-inflammatory, and targeted intervention paradigms, frames disease as a problem of invasion or excess that can be corrected through suppression or eradication. While such approaches have demonstrated clear effectiveness in acute and well-defined pathological contexts, their limitations become increasingly visible when applied to chronic physiological states unfolding within symbiotic biological systems (Medzhitov, 2008; Nathan and Ding, 2010; Hotamisligil, 2017).

The structural patterns identified in the present synthesis suggest that host–exogenous biological interaction does not operate as a transient disturbance of an otherwise autonomous host system. Instead, the literature repeatedly documents configurations in which microbial, viral, and environmental genomic signals participate in shaping host regulatory behavior across immune, metabolic, barrier, and neuroendocrine domains. These observations align with the broader biological understanding that the human organism is not a closed entity periodically breached by external agents, but a permanently symbiotic system in which host and exogenous genomic populations coexist and co-regulate physiological function (Douglas, 2010; Human Microbiome Project Consortium, 2012; McFall-Ngai et al., 2013; Liang and Bushman, 2021; Tiamani et al., 2022).

Within symbiotic systems, elimination of a biological participant does not necessarily restore a neutral baseline. Unlike acute pathogenic invasion, where removal of an agent may terminate the perturbation, chronic physiological configurations frequently persist after the initiating trigger has been suppressed. The literature examined in the present study repeatedly describes situations in which inflammatory tone, immune thresholds, metabolic routing, or barrier permeability remain altered even when microbial abundance changes or pathogen clearance is achieved. These observations indicate that biological systems reorganize around perturbations rather than simply reverting to a previous equilibrium (Angriman et al., 2014; Barbara et al., 2019; Annavajhala et al., 2020; Banerjee et al., 2026).

This behavior is consistent with principles of symbiotic organization widely documented in ecology and evolutionary biology. In complex biological systems composed of multiple interacting genomes, stability emerges not from the absence of interaction but from the regulation of interaction. Symbiosis therefore represents a structural condition of living systems rather than an accessory feature of them. The persistence of coherent yet clinically adverse physiological states can thus be interpreted as the stabilization of adaptive configurations within a symbiotic environment rather than as simple failure of host regulatory mechanisms (Douglas, 2010; Noble, 2012; McFall-Ngai et al., 2013; Theis et al., 2016).

Importantly, recognizing the symbiotic organization of the human system does not negate the clinical necessity of eradication in specific contexts. Acute infections, toxic exposures, and clearly defined pathogenic invasions often require decisive suppression to prevent catastrophic loss of host function. However, extending eradication-centered reasoning to chronic physiological conditions may constitute a conceptual limitation. When pathological states emerge from stabilized host–exogenous interaction regimes, elimination of a single biological participant may disrupt components of the system without necessarily altering the underlying configuration that maintains the state (Borba et al., 2019; Iliev and Cadwell, 2021; Jensen et al., 2024).

Within this perspective, chronic physiological conditions may reflect not the persistence of a single agent but the stabilization of an ecological and regulatory arrangement in which host and exogenous biological signals remain dynamically integrated. Understanding how such configurations emerge and persist therefore requires moving beyond agent-centered models toward frameworks capable of describing how symbiotic systems organize their regulatory priorities over time (Simon, 1977; Kauffman, 1993; Gigerenzer and Gaissmaier, 2011).

At this point, the interpretation of host–exogenous interaction must be reframed within the broader context of symbiotic biological organization. If physiological regulation emerges within a multigenomic ecological environment in which multiple biological actors continuously interact, then persistent physiological states cannot be understood solely through molecular pathways or isolated biological agents. Instead, they reflect how interacting genomic systems stabilize regulatory priorities within a shared physiological environment. This transition from agent-centered interpretation to symbiotic systems reasoning establishes the conceptual threshold from WP4 structural plausibility assessment toward the systems-level integration formalized in the subsequent sections under WP5.

### 4.2 Symbiotic multigenomic organization of living systems

The structural interpretation emerging from the present synthesis is consistent with a broader biological principle: living organisms rarely function as genetically autonomous entities. Across multiple branches of biology—including ecology, parasitology, evolutionary biology, and microbiology—it is well established that physiological organization frequently emerges from symbiotic interactions among multiple genomic systems (Douglas, 2010; Gilbert et al., 2012; McFall-Ngai et al., 2013; Theis et al., 2016).

In many biological contexts, host organisms coexist with persistent exogenous genomic populations that influence metabolism, immunity, development, and behavior. These relationships range from obligate mutualisms to parasitic interactions, yet in all cases the resulting biological system represents a composite ecological structure rather than a single-genome organism (Douglas, 2010; McFall-Ngai et al., 2013; Liang and Bushman, 2021; Sender et al., 2016).

Numerous non-human systems illustrate this principle. Parasite-induced behavioral modification in rodents infected with *Toxoplasma gondii* demonstrates how relatively small exogenous genomes can induce durable alterations in host neural responses without immediately compromising organismal viability (Webster and McConkey, 2010; Vyas, 2015; Gilbert et al., 2012). Similarly, fungal parasites such as *Ophiocordyceps* are capable of redirecting insect locomotor behavior toward configurations that favor fungal reproduction, while the host organism maintains physiological coherence long enough to complete the parasite’s life (Hughes et al., 2011; Bekker et al., 2014).

Other examples highlight symbiosis in its cooperative form. Termite digestion depends on complex microbial consortia capable of degrading cellulose, a metabolic capacity absent from the host genome itself (Ohkuma, 2008; Brune, 2014). Coral–algal associations similarly demonstrate that host organisms can actively regulate symbiotic partnerships in response to environmental conditions, expelling symbionts when ecological constraints destabilize energetic balance (Muscatine and Porter, 1977; Baker et al., 2008).

Viral systems provide a particularly clear boundary condition for this phenomenon. During infection, relatively compact viral genomes can redirect host transcriptional and metabolic machinery toward viral replication while preserving short-term cellular viability (Handley, 2016; Liang and Bushman, 2021). In such contexts, host cellular processes remain coherent but are temporarily reorganized around the life cycle of an exogenous genomic system.

Taken together, these observations indicate that biological systems frequently stabilize around multigenomic configurations in which host and exogenous genomes coexist within shared metabolic and signaling environments. Rather than representing anomalies, such arrangements constitute common organizational strategies in living systems (Kauffman, 1993; Gilbert et al., 2012; Theis et al., 2016).

When viewed in this broader biological context, the concept of a multigenomic human organism becomes less radical and more consistent with established principles of symbiotic biology. The human body hosts dense microbial ecosystems, persistent viral populations, and diverse environmental genomic signals that continuously interact with host regulatory processes. Human physiology therefore unfolds within a symbiotic genomic environment rather than within a purely host-derived regulatory space.

Recognizing this symbiotic multigenomic organization provides the conceptual foundation necessary to interpret the membrane-level integration processes described in the following section.

### 4.3 The cellular membrane as the integrative interface of multigenomic systems

The structural patterns identified in the present synthesis consistently converge toward a common biological interface: the cellular membrane. Across the retained corpus, mechanisms involved in host–exogenous interaction repeatedly appear mediated through processes that operate at or near membrane-level signaling platforms. Barrier permeability, receptor-mediated sensing, redox-sensitive signaling, metabolite transport, and nucleic acid recognition all involve regulatory events that occur at membrane interfaces or within membrane-associated signaling environments (Pike, 2006).

This convergence suggests that the cellular membrane represents a central operational interface through which host and exogenous biological signals become integrated within physiological systems. While microbial populations, viral elements, and environmental metabolites originate outside host cellular genomes, their regulatory influence typically becomes physiologically relevant only once interpreted through membrane-associated signaling structures. In this sense, the membrane does not merely delimit the cell; it functions as a dynamic regulatory platform where ecological signals are translated into intracellular biological responses (Changede and Sheetz, 2017; Shi et al., 2018; Ruffinatti et al., 2023).

The literature examined in this study repeatedly documents membrane-associated mechanisms through which exogenous biological information is interpreted. Pattern-recognition receptors embedded in epithelial and immune cell membranes detect microbial molecular patterns and initiate signaling cascades that recalibrate immune thresholds (Medzhitov, 2008; Netea et al., 2016; Yang et al., 2019). Transporters and receptor systems regulate the cellular uptake of microbial metabolites such as short-chain fatty acids, bile acid derivatives, and tryptophan metabolites, thereby linking microbial ecology to host metabolic regulation (Valdes et al., 2018; Ahmed et al., 2022; Valencia et al., 2025). Similarly, viral RNA sensing mechanisms operate through membrane-associated complexes that initiate antiviral signaling and transcriptional responses (Watanabe et al., 2011; Perego et al., 2018; Obermann et al., 2022).

Importantly, these interactions do not occur within a static physicochemical environment. Membrane behavior is itself strongly influenced by local microenvironmental conditions, including pH gradients, redox balance, oxygen tension, lipid composition, and proton dynamics (Abrescia et al., 2020). These variables shape receptor responsiveness, signal amplification, and the energetic conditions under which intracellular signaling pathways operate (Sun et al., 2009; Sahl et al., 2017). Consequently, the cellular membrane can be understood as the site where ecological signals originating from exogenous genomic systems intersect with the physicochemical constraints governing host cellular physiology.

Within multigenomic biological systems, this interface acquires particular significance. Host cells operate within environments populated by persistent microbial communities and exposed to diverse exogenous molecular signals. The membrane therefore becomes the locus where host regulatory programs continuously interpret, filter, and respond to ecological inputs derived from these exogenous genomic populations. Rather than acting as passive recipients of microbial influence, membrane-associated signaling networks actively shape how such signals are weighted within cellular decision processes (Liu et al., 2016; Abate et al., 2020; Bibby et al., 2020).

The stabilization mechanisms identified in the Results section—barrier remodeling, immune recalibration, metabolic reprogramming, and neuroendocrine coupling—can all be traced to regulatory processes that depend on membrane-level interpretation of environmental signals. In this sense, these mechanisms do not represent independent physiological phenomena but different expressions of a common interface through which multigenomic biological interaction becomes operational within host physiology.

Recognizing the membrane as this integrative interface provides a coherent biological explanation for the structural asymmetry identified in the literature. Functional host–microbe associations are extensively documented because they manifest at system-level physiological outputs. In contrast, the regulatory interfaces through which these interactions are operationalized—membrane signaling environments, redox-modulated receptor behavior, and nucleic acid sensing complexes—remain comparatively under-integrated within broader conceptual frameworks (Simons and Gerl, 2010; He et al., 2018; Zhou et al., 2022).

Viewing host–exogenous biological interaction through the lens of membrane-level integration therefore allows the diverse mechanisms documented across the literature to be understood within a common organizational architecture. Within such a framework, microbial signals, environmental inputs, and host regulatory processes converge at membrane interfaces, where ecological information is translated into intracellular signaling dynamics that ultimately shape systemic physiological behavior.

Importantly, when membrane-level signaling interfaces repeatedly bias cellular responses under stable ecological and physicochemical conditions, these processes may progressively reshape the energetic landscape in which regulatory dynamics unfold. Under such circumstances, physiological organization may converge toward relatively stable configurations of signaling and metabolism, a phenomenon conceptually consistent with attractor dynamics described in complex biological systems (Noble, 2012; Project Management Institute, 2019).

### 4.4 Bioenergetic attractor states in multigenomic systems

Biological systems rarely operate as linear chains of molecular causality. Instead, they tend to organize around relatively stable configurations of energy flow, signaling integration, and regulatory interaction. In complex systems theory, such configurations are commonly described as attractor states: dynamically stable regimes toward which a system converges under specific ecological and physicochemical constraints (Kauffman, 1993).

Within a multigenomic biological organism, attractor dynamics provide a useful conceptual framework for interpreting the persistence of physiological configurations that emerge from host–exogenous interaction. Host cells operate within environments shaped not only by endogenous genetic programs but also by the metabolic, chemical, and signaling outputs of resident microbial and viral populations (Valencia et al., 2025). These interactions collectively define the physicochemical landscape within which cellular regulatory systems operate.

In energetically favorable configurations, cellular metabolism, mitochondrial respiration, redox oscillation, and signaling responsiveness remain flexible and adaptive. Such states are characterized by broad oscillatory ranges across metabolic, immune, and neuroendocrine axes, allowing biological systems to respond efficiently to changing environmental conditions (Rich and Maréchal, 2010). Within these regimes, microbial symbiosis remains integrated under host-prioritized functional organization, and physiological processes maintain high decisional plasticity (Valdes et al., 2018; Wiertsema et al., 2021).

However, sustained ecological pressures—including persistent microbial dysbiosis, chronic inflammatory signaling, environmental stressors, altered nutrient landscapes, or repeated metabolic perturbations—can progressively reshape the physicochemical environment in which host cells operate (Nathan and Ding, 2010; Franceschi et al., 2017; Hotamisligil, 2017). Under these conditions, cellular regulatory systems may converge toward alternative energetic configurations that remain internally coherent yet operate under narrower oscillatory ranges (Alon, 2007; Brown, 2008).

These stabilized configurations can be interpreted as displaced bioenergetic attractor states. Rather than representing chaotic dysfunction, such states constitute energetically sustainable regimes in which metabolic routing, immune signaling thresholds, barrier permeability, and neuroendocrine responses become recalibrated to maintain system-level coherence under altered environmental conditions.

Importantly, attractor stabilization does not require structural damage or irreversible molecular failure. Changes in mitochondrial efficiency, redox balance, membrane signaling thresholds, and microenvironmental conditions can be sufficient to bias cellular decision processes toward particular regulatory pathways. Over time, these shifts may progressively reinforce the stability of the new configuration through feedback processes involving metabolic adaptation, immune recalibration, and epigenetic modulation.

A defining characteristic of attractor dynamics is hysteresis. Once biological systems transition into a stabilized configuration, removal of the initiating perturbation does not necessarily restore the previous physiological regime. The system may remain within the basin of attraction defined by the new configuration because the energetic and regulatory landscape has already been reorganized.

Within the context of host–exogenous biological interaction, this phenomenon provides a plausible explanation for the persistence of physiological states that remain stable despite changes in microbial abundance, pathogen clearance, or targeted intervention. The attractor framework therefore offers a conceptual bridge between ecological interaction, membrane-level regulatory processes, and long-term physiological organization within multigenomic systems.

### 4.5 Membrane-level signal weighting as a basis for understanding symbiotic multigenomic relationships

The structural synthesis developed in the preceding sections supports a more specific interpretative proposition. If the human organism is understood as a symbiotic multigenomic system, continuously exposed to microbial metabolites, exogenous nucleic acids, environmental inputs, pharmacological signals, and host-derived regulatory molecules, then physiological organization cannot be explained solely by the isolated action of any single signal source. Rather, biological regulation appears to depend on how combinations of signals are integrated within shared regulatory interfaces.

Within this framework, the cellular membrane emerges as more than a selective boundary or passive receptor platform. The evidence reviewed here suggests that it functions as an active weighting interface through which multiple extracellular influences are differentially interpreted, routed, and translated into intracellular responses. The biological relevance of a given signal therefore depends not only on its presence, but also on the membrane-associated context in which it is received, combined with other inputs, and converted into downstream regulatory consequences (Changede and Sheetz, 2017; Ruffinatti et al., 2023).

This interpretation is especially consistent with the recurrent convergence identified across barrier modulation, immune recalibration, metabolic reprogramming, neuroendocrine coupling, and physicochemical microenvironmental conditioning. These processes indicate that cellular responses are not determined by linear stimulus–response logic alone. Instead, they suggest that host cells continuously operate within combinatorial fields of influence in which membrane-associated signaling architecture contributes to determining which inputs acquire effective physiological priority.

In a symbiotic multigenomic organism, such weighting processes become particularly significant. Host cells coexist within an environment that is not purely host-derived but ecologically shared with persistent exogenous genomic populations and their molecular outputs. Under these conditions, membrane-level signal integration may progressively bias certain regulatory configurations over others, especially when reinforced by stable microenvironmental conditions and bioenergetic attractor dynamics (Kauffman, 1993; Sterling, 2012; Verdin, 2015). In symbiotic systems, these interactions may help explain governance-like regimes that have been repeatedly documented across biological systems in nature. Over time, recurrent signal-weighting patterns may contribute to the stabilization of coherent physiological regimes that reflect forms of symbiotic governance (Noble, 2012; Relman, 2012).

At this point, the concept of physiological governance becomes analytically useful. In the present framework, governance does not imply conscious control, intentionality, or centralized command. Rather, it refers to the stabilized hierarchical organization through which regulatory influence is distributed and weighted within a multigenomic biological system. Clinical manifestations may therefore be interpreted not merely as downstream outputs of isolated dysfunctions, but as organism-level expressions of how membrane-level signal weighting, ecological interaction, and bioenergetic stabilization become organized over time.

**Figure 4:**
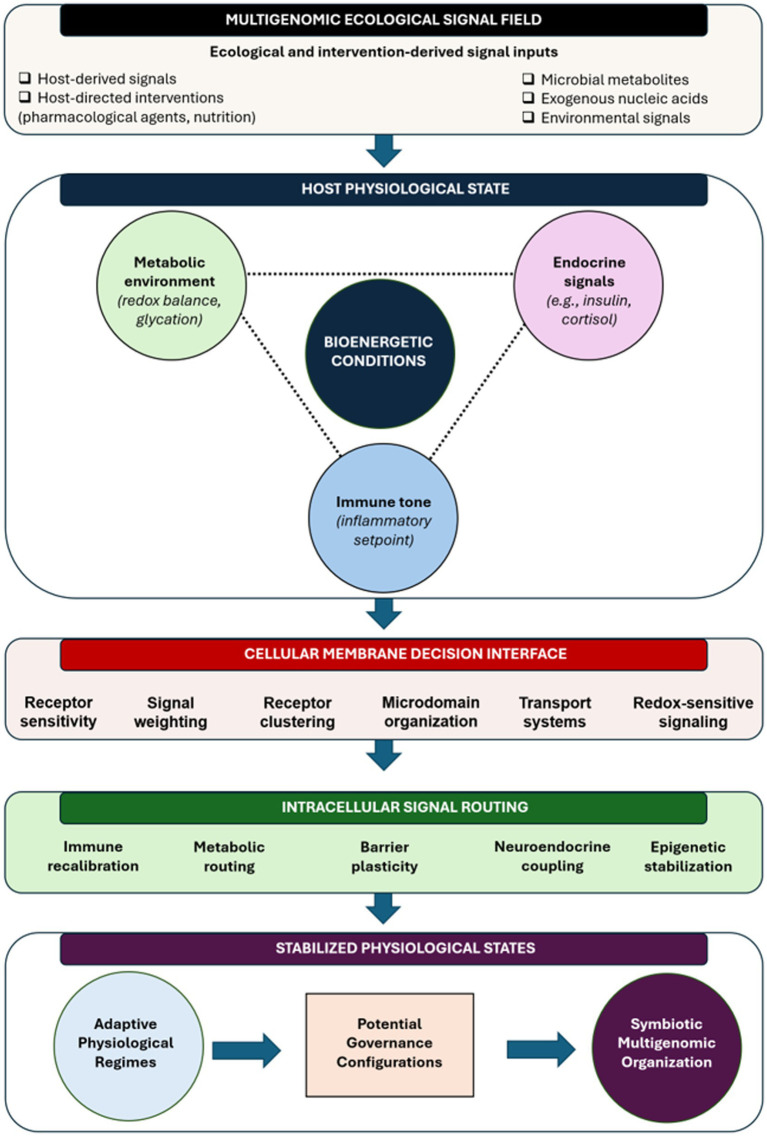
Multigenomic signal integration and stabilization of physiological regimes. The figure illustrates a conceptual model describing how physiological organization may emerge within a symbiotic multigenomic system. Multiple classes of ecological and intervention-derived signals—including host-derived regulatory molecules, microbial metabolites, exogenous nucleic acids, environmental exposures, and host-directed inputs such as nutrition or pharmacological agents—converge within a shared biochemical environment. These signals interact with the prevailing human physiological state, defined by metabolic conditions, endocrine signaling, immune tone, and underlying bioenergetic constraints. Within this physiological context, the cellular membrane functions as a decision interface in which extracellular signals are sensed, integrated, and differentially weighted through receptor sensitivity, microdomain organization, transport systems, and redox-sensitive signaling platforms. This integration determines how signals are routed into intracellular regulatory pathways. Downstream signaling processes—including immune recalibration, metabolic routing, barrier plasticity, neuroendocrine coupling, and epigenetic stabilization—may progressively reinforce coherent regulatory configurations across cellular systems. Over time, these dynamics can stabilize physiological attractor states, giving rise to adaptive physiological regimes within multigenomic biological environments.

From this perspective, symptoms, chronic inflammatory states, metabolic rigidity, neuroendocrine bias, and persistent maladaptive phenotypes do not necessarily indicate absence of regulation. They may instead reflect the presence of regulation operating under displaced yet coherent priorities. The clinical phenotype thus becomes intelligible as an emergent expression of physiological governance shaped by the interaction between host biology, exogenous genomic influence, and membrane-level decisional architecture, as conceptually illustrated in Figure 4.

### 4.6 Evaluation of the framework using SMART and FINER criteria

To ensure that the interpretative architecture proposed in this study remains scientifically tractable and relevant for future investigation, the framework was examined using established SMART (Doran, 1981) and FINER (Hulley et al., 2007) criteria commonly applied in biomedical research design.

This evaluation was not intended as empirical validation but as a structured appraisal of whether the conceptual architecture generates research questions that are specific, testable, feasible, and methodologically meaningful. The resulting assessment is summarized in Table 2.

**Table 2:** Evaluation of the proposed framework according to SMART and FINER criteria.

Criterion	Application to the proposed framework	Example of testable implication
SMART framework		
Specific	Identifies membrane-associated signaling interfaces as the locus of multigenomic signal integration	Measurement of receptor clustering, metabolite transporters, and nucleic acid sensing complexes
Measurable	Uses experimentally accessible variables including redox tone, membrane signaling thresholds, and mitochondrial bioenergetics	Redox assays, mitochondrial respiration analysis, membrane receptor activity
Achievable	Compatible with existing microbiome, cell signaling, and metabolic experimental platforms	In vitro microbiome-host co-culture models
Relevant	Addresses persistent physiological states in chronic inflammatory, metabolic, and neuropsychiatric conditions	Investigation of attractor stabilization in chronic disease
Time-bound	Enables longitudinal evaluation of stabilization dynamics	Temporal analysis of host–microbe interaction regimes
FINER framework		
Feasible	Compatible with current microbiome, physiology, and systems biology methodologies	Multi-omics integration
Interesting	Integrates microbiome ecology with membrane signaling and systems physiology	Cross-domain research programs
Novel	Introduces membrane-centered architecture for multigenomic physiological organization	Framework for hypothesis generation
Ethical	Does not prescribe interventions or experimental manipulation in humans	Conceptual and analytical framework
Relevant	Addresses unresolved stability of chronic physiological states	Translational research on host–microbe systems

## 5 Discussion

The present study does not propose a new molecular mechanism or isolated explanatory pathway. Rather, its principal contribution lies in reorganizing convergent evidence on host–exogenous biological interaction into a systems-level interpretative architecture capable of contextualizing long-term physiological stabilization within multigenomic biological environments.

Across WP2–WP5, the structured synthesis consistently indicated that host–microbe interaction cannot be fully interpreted through isolated domain-specific mechanisms alone. Instead, recurrent cross-domain correspondences involving barrier modulation, immune recalibration, metabolic routing, neuroendocrine integration, and membrane-associated signaling suggest that physiological organization may emerge through coordinated regulation across multiple biological interfaces operating within shared ecological and physicochemical conditions.

Within this perspective, the discussion that follows explores the organizational implications of these convergence patterns, particularly regarding membrane-level signal integration, stabilization dynamics, and the persistence of long-term physiological configurations in multigenomic systems. These interpretations should be understood as structured systems-level abstractions derived from cross-domain evidence convergence rather than as definitive causal explanations of biological regulation.

### 5.1 Reinterpreting host–microbiome interaction in multigenomic systems

The expansion of microbiome research has substantially transformed contemporary understanding of human physiology. Accumulating evidence demonstrates that microbial communities participate in immune regulation, metabolic homeostasis, epithelial barrier integrity, and neuroendocrine signaling across multiple physiological domains (Human Microbiome Project Consortium, 2012; Qin et al., 2010; Cryan et al., 2019; Fan and Pedersen, 2021). Nevertheless, host–microbiome interaction continues to be predominantly interpreted through host-centered frameworks in which microbial signals function primarily as modulators of otherwise host-determined physiological processes (Relman, 2012; Douglas, 2010).

The systems-level synthesis developed in the present study suggests that this conceptual framing may incompletely represent the degree of biological integration observed across the retained literature. Rather than operating as occasional external modifiers, microbial, viral, and environmental genomic signals recurrently appear as components of the ecological and biochemical environments within which physiological regulation occurs (Gilbert et al., 2012; McFall-Ngai et al., 2013). Within this interpretative perspective, host physiology may be more coherently understood as unfolding within a symbiotic multigenomic system characterized by continuous interaction across shared regulatory interfaces.

Importantly, symbiotic organization does not necessarily imply optimization at the host level. Biological systems frequently stabilize coherent configurations that preserve overall functional organization while operating under ecological or physiological priorities that may not fully align with maximal host adaptability (Kauffman, 1993; Noble, 2012). In this context, the concept of physiological governance becomes analytically useful for describing how regulatory influence may become stabilized and hierarchically distributed within multigenomic biological environments (Gaspary et al., 2026).

From this perspective, persistent inflammatory tone, recurrent infection despite pathogen clearance, heterogeneous responses to nutritional or metabolic interventions, and resistance to targeted therapeutic strategies may be interpreted not solely as isolated mechanistic dysfunctions, but as manifestations of stabilized regulatory configurations emerging from distributed signal integration across symbiotic biological systems.

### 5.2 Membrane-level signal integration as significant biological decision Interface

The interpretative framework developed in the present study suggests that membrane-associated signaling environments may represent a central regulatory substrate through which multigenomic interaction becomes operational within cellular physiology. Rather than functioning exclusively as structural barriers or passive receptor platforms, cellular membranes may be more coherently interpreted as dynamic integration interfaces in which extracellular signals are continuously filtered, weighted, and translated into context-dependent intracellular responses.

Within classical cell biology, regulatory organization is frequently attributed primarily to intracellular genetic programs and signaling cascades triggered by receptor activation (Brown and London, 2000; Pike, 2006). However, the convergence patterns identified across the retained literature suggest that membrane-associated organization itself may substantially influence how signaling relevance is established under physiological conditions. Microbial metabolites, viral molecular signals, endocrine mediators, inflammatory factors, and physicochemical microenvironmental conditions repeatedly converge within membrane-associated signaling environments before downstream intracellular responses are activated (Vidakovics et al., 2010; Changede and Sheetz, 2017; Ruffinatti et al., 2023).

Within this perspective, the membrane becomes analytically relevant not merely because it receives extracellular information, but because it may regulate which signals acquire sufficient decisional weight to influence subsequent physiological organization. Barrier plasticity, immune recalibration, metabolic routing, and neuroendocrine coupling recurrently intersect with membrane-associated sensing systems whose responsiveness depends on local physicochemical conditions such as lipid composition, redox balance, pH gradients, and proton dynamics (Wang, 2012). These variables collectively shape signaling thresholds, amplification dynamics, and persistence of intracellular responses.

This interpretation provides a plausible systems-level mechanism through which multigenomic interaction may contribute to stabilization of long-term physiological configurations. Under sustained ecological and physiological conditions, repeated membrane-level signal prioritization may progressively reinforce specific regulatory pathways through metabolic adaptation, immune recalibration, and epigenetic stabilization processes (Noble, 2012; Franceschi et al., 2017; Hotamisligil, 2017).

In this context, membrane-associated signaling architecture may be interpreted not simply as a receptor surface, but as a dynamic biological decision interface through which regulatory priorities become organized within multigenomic physiological systems.

### 5.3 Human physiological state as a determinant of membrane-level signal integration

An important implication of the membrane-centered interpretative framework developed in the present study is that signal integration does not occur under physiologically neutral conditions. Membrane-associated signaling environments operate within continuously changing biochemical, endocrine, metabolic, and inflammatory contexts that directly influence receptor sensitivity, signaling thresholds, and intracellular routing dynamics.

Within this perspective, the biological relevance of extracellular signals may depend not only on ligand identity or concentration, but also on the physiological state of the host system in which these signals are interpreted. Endocrine and metabolic variables such as cortisol signaling, insulin regulation, substrate availability, redox balance, and oxidative stress are all capable of modifying membrane-associated signaling behavior through alterations in lipid organization, receptor conformation, and intracellular responsiveness (Hotamisligil, 2017; Yoshino et al., 2018; Abrescia et al., 2020; Covarrubias et al., 2021).

Similarly, persistent inflammatory tone may substantially reshape membrane-associated sensing environments. Chronic low-grade inflammation has been repeatedly associated with recalibration of immune receptor thresholds, modification of cytokine signaling networks, and altered responsiveness of host–microbe interaction systems (Nathan and Ding, 2010; Franceschi et al., 2017; Netea et al., 2020). Under such conditions, membrane-associated signaling platforms may progressively favor particular regulatory pathways while reducing responsiveness to alternative physiological inputs.

Taken together, these observations suggest that membrane-level signal integration is strongly conditioned by the prevailing physiological organization of the host system itself. Microbial metabolites, viral molecular signals, endocrine mediators, inflammatory factors, and environmental biochemical inputs do not operate independently, but converge within shared regulatory environments whose responsiveness depends on the current physiological state.

This perspective may help explain why host–exogenous biological interaction cannot be fully interpreted through microbial variables alone. Similar microbial signals may generate substantially different physiological consequences depending on the endocrine, metabolic, inflammatory, and bioenergetic conditions within which they are processed (Relman, 2012; Wiertsema et al., 2021).

Within multigenomic biological systems, membrane-associated routing architecture therefore provides a plausible mechanistic bridge linking ecological exposure, physiological state conditioning, and long-term stabilization of organism-level regulatory behavior.

### 5.4 Stabilization of physiological regimes in multigenomic systems

The interpretative framework developed throughout the preceding sections suggests that physiological organization in multigenomic systems may not be fully understood through isolated molecular pathways alone. Instead, regulatory behavior appears to emerge from continuous interaction between ecological signals, membrane-level signal integration, and the prevailing physiological state within which these signals are interpreted.

When such interactions recur under relatively stable ecological and physiological conditions, cellular responses may progressively converge toward reinforced signaling configurations. Membrane-associated sensing systems, metabolic routing pathways, immune thresholds, and neuroendocrine regulatory loops begin to operate within increasingly self-reinforcing patterns of responsiveness. Over time, metabolic adaptation, immune recalibration, and epigenetic modulation may further stabilize these configurations by reinforcing repeatedly activated regulatory pathways (Yoshino et al., 2018).

Such stabilization dynamics are widely recognized features of complex adaptive systems. Rather than fluctuating indefinitely between alternative responses, biological systems tend to converge toward relatively stable configurations capable of remaining energetically sustainable within prevailing environmental conditions (Kauffman, 1993; Alon, 2007; Noble, 2012).

Within multigenomic physiological systems, these stabilized configurations may emerge from persistent interaction between host-derived regulatory programs and exogenous genomic signals operating within shared biochemical and physicochemical environments. Microbial metabolites, endocrine mediators, inflammatory signals, and environmental biochemical inputs collectively shape the regulatory landscape within which cellular signaling decisions occur. When particular combinations of these inputs repeatedly favor specific regulatory pathways, physiological organization may progressively stabilize around those pathways.

Importantly, such stabilization does not necessarily imply structural damage or irreversible dysfunction. Physiological systems may remain internally coherent while operating within regulatory regimes that differ from those typically associated with optimal host adaptability. Under such conditions, metabolic routing, immune thresholds, barrier permeability, and neuroendocrine signaling may remain coordinated while operating within narrower oscillatory ranges than those observed in highly adaptive physiological states.

This perspective offers a plausible explanation for the persistence of physiological configurations that remain stable even when initiating triggers partially resolve or change over time. Because stabilization emerges from repeated interaction between ecological signals and prevailing physiological conditions, modification of isolated pathways or individual signal sources may not immediately reorganize the broader regulatory regime sustaining the state.

Within multigenomic organisms, long-term physiological behavior may therefore reflect stabilization of coherent regulatory configurations emerging from distributed signal integration across ecological, metabolic, immune, and bioenergetic domains. Understanding how such regimes stabilize, persist, and transition across physiological contexts may therefore represent a central systems-level challenge for future host–exogenous interaction research.

### 5.5 Symbiotic governance regimes in multigenomic physiology

The stabilization dynamics discussed in the preceding sections suggest that long-term physiological organization in multigenomic systems may be more coherently interpreted through the concept of governance regimes. Within this framework, governance does not imply intentionality, centralized control, or autonomous agency. Rather, it refers to the stabilized hierarchical organization through which regulatory influence becomes distributed across interacting biological systems operating under shared ecological and physicochemical constraints (Simon, 1977; Alon, 2007; Gaspary et al., 2026).

Within multigenomic physiological environments, membrane-associated signaling interfaces continuously integrate microbial metabolites, endocrine mediators, inflammatory signals, environmental biochemical inputs, and host-derived regulatory molecules within the same signaling field (He et al., 2018; Ruffinatti et al., 2023). Under sustained ecological and physiological conditioning, repeated patterns of signal prioritization may progressively reinforce particular regulatory configurations governing metabolic behavior, immune responsiveness, neuroendocrine regulation, and barrier dynamics.

When such configurations become sufficiently stabilized, they may function as governance regimes: coherent physiological organizations in which regulatory priorities remain persistently biased toward specific adaptive patterns. Importantly, these regimes do not arise from dominance of isolated biological agents or pathways, but from cumulative interaction among multiple signaling systems operating within shared physiological environments (Gilbert et al., 2012; Theis et al., 2016).

This perspective does not imply that governance regimes are inherently pathological or beneficial. In complex biological systems, stability frequently reflects preservation of systemic coherence under prevailing environmental constraints rather than optimization of host-level adaptability (Kauffman, 1993; Noble, 2012). Consequently, physiological systems may stabilize around configurations that maintain internal organization while simultaneously sustaining inflammatory bias, metabolic rigidity, altered neuroendocrine responsiveness, or reduced adaptive flexibility (Franceschi et al., 2017; Hotamisligil, 2017).

Within this interpretative framework, persistent inflammatory tone, recurrent infection despite pathogen clearance, heterogeneous responses to nutritional interventions, and resistance to targeted therapeutic strategies may be understood not exclusively as manifestations of isolated dysfunction, but as expressions of stabilized regulatory regimes operating within multigenomic physiological environments.

Understanding host physiology through the lens of governance regimes therefore expands the conceptual space through which long-term physiological organization may be interpreted. Rather than focusing exclusively on isolated molecular pathways or individual biological agents, this perspective emphasizes how regulatory influence may become stabilized across interacting genomic systems operating within shared ecological and physiological conditions.

### 5.6 Chronic diseases as stabilized adaptive physiological states

The interpretative framework developed throughout this discussion supports reconsideration of how persistent pathological conditions may be conceptualized within multigenomic biological systems. If host physiology operates through continuous integration of host-derived and exogenous signals within membrane-associated regulatory interfaces, and if such integrations progressively stabilize into coherent regulatory regimes, then long-term physiological states commonly described as chronic diseases may reflect stabilized adaptive configurations rather than simple regulatory failure, consistent with previous systems-level organizational interpretations of chronic physiological stability (Gaspary et al., 2026).

Within this perspective, chronic physiological conditions may emerge when regulatory systems converge toward signaling configurations that remain energetically sustainable under prevailing ecological and biochemical constraints. Such configurations may preserve systemic coherence while operating within narrower adaptive ranges, characterized by persistent inflammatory tone, altered metabolic routing, modified neuroendocrine signaling, or reduced physiological plasticity.

Importantly, this interpretation does not imply that chronic diseases represent optimal biological states, nor does it negate the well-established mechanistic pathways underlying specific pathologies. Rather, it suggests that persistent pathological phenotypes may arise when regulatory systems stabilize around configurations that remain internally coherent within multigenomic ecological environments (Lozupone et al., 2012; Relman, 2012; Valencia et al., 2025).

This perspective may help explain why many chronic conditions display resistance to targeted interventions directed at single molecular pathways or biological agents. When physiological states emerge from distributed regulatory regimes shaped by membrane-level signal integration and ecological interaction, modification of isolated components may not be sufficient to alter the broader configuration that sustains the state.

Understanding chronic disease through the lens of stabilized adaptive organization may therefore provide a systems-level framework for investigating how physiological states emerge, persist, and transition across multigenomic biological environments.

### 5.7 Interventions are not host-specific in multigenomic systems

The multigenomic interpretation developed throughout this study also raises an important question regarding the biological scope of physiological interventions. If human physiology operates within a symbiotic system characterized by continuous interaction between host-derived and exogenous genomic signals, then interventions directed toward the host organism may not act exclusively on host cellular processes (Douglas, 2010; McFall-Ngai et al., 2013).

Many forms of physiological modulation—including dietary change, pharmacological treatment, supplementation, metabolic intervention, and environmental conditioning—alter biochemical environments shared simultaneously by host cells and resident microbial communities. Under such conditions, substrates, metabolites, signaling molecules, and energetic resources introduced into the system become accessible not only to host regulatory pathways but also to exogenous genomic populations occupying the same ecological niches (Valdes et al., 2018; Myhrstad et al., 2020; Ahmed et al., 2022).

Within a symbiotic multigenomic framework, this observation suggests that interventions traditionally conceptualized as host-directed may also function as ecological modulators of the broader physiological system. Alterations in nutrient availability, redox balance, metabolic substrates, hormonal signaling, or physicochemical conditions may simultaneously influence host regulatory behavior and microbial ecological organization (Wiertsema et al., 2021; Valencia et al., 2025).

Importantly, this perspective does not imply loss of host regulatory autonomy, nor does it suggest that exogenous genomic systems respond uniformly to intervention. Rather, it highlights that physiological modulation occurs within shared biochemical environments in which multiple biological systems coexist and dynamically interact.

The present study did not attempt to evaluate these interactions empirically. Nevertheless, if physiological organization emerges within multigenomic ecological systems, then interventions cannot be assumed to act exclusively upon host biology in isolation. Understanding how physiological modulation reorganizes the shared biochemical environment of host and exogenous genomic systems may therefore represent an important systems-level frontier for future translational research.

### 5.8 Scope boundaries and conceptual limitations

The interpretative framework proposed in this study is intentionally conceptual in scope and several boundaries should therefore be acknowledged. The objective of the present synthesis was not to establish direct causal relationships between specific microbial taxa, viral agents, or exogenous genomic elements and defined clinical diseases. Rather, the analysis sought to examine how the existing literature is structurally organized across different levels of biological interaction and to explore how these patterns may inform broader interpretations of physiological organization in multigenomic systems.

Accordingly, the concept of physiological governance introduced in this discussion should not be interpreted as a measurable biological variable or a diagnostic category. Governance is used here as an organizational descriptor intended to capture how regulatory influence may become stabilized within interacting genomic systems operating under shared ecological and biochemical constraints. The framework therefore does not propose biomarkers, therapeutic targets, or clinical classifications based on governance states.

It is also important to emphasize that the multigenomic interpretation advanced in this work does not imply deterministic control of host physiology by microbial or exogenous genomic systems. Host-derived genetic programs, metabolic regulation, immune responses, endocrine signaling, and tissue-specific constraints remain central determinants of physiological organization. The framework instead proposes that these host regulatory systems operate within ecological environments shaped by persistent exogenous genomic signals, and that the resulting physiological outcomes emerge from the integration of these interacting influences.

Similarly, the interpretation of chronic physiological states as stabilized adaptive configurations should not be understood as a claim that all diseases arise from multigenomic ecological interaction. Numerous pathological processes are driven primarily by genetic mutations, acute infections, toxic exposures, or structural injury. The present framework simply suggests that in many chronic contexts, long-term physiological behavior may reflect stabilization of regulatory regimes emerging from distributed signal integration rather than isolated pathway disruption.

Another important limitation concerns the methodological nature of the present study. Because the analysis prioritized structural mapping and cross-domain synthesis, it did not attempt to quantify effect sizes or perform meta-analytical comparisons between studies. Instead, the synthesis focused on identifying recurrent mechanistic patterns and structural asymmetries within the literature. Future empirical research will be necessary to test the hypotheses generated by this framework and to determine under which conditions membrane-level signal integration and multigenomic interaction contribute to the stabilization of physiological regimes.

Finally, the framework proposed here should be understood as a heuristic model intended to guide future investigation rather than as a definitive explanation of host–exogenous biological interaction. Its primary contribution lies in reorganizing a fragmented body of literature into a coherent interpretative architecture capable of generating new research questions regarding the long-term organization of physiological states in multigenomic biological systems.

## 6 Conclusion

The structured synthesis conducted in this study suggests that the current evidentiary landscape of host–exogenous biological interaction is characterized by a marked asymmetry. Functional associations between microbial activity and host physiological processes are extensively documented across immune, metabolic, barrier, and neuroendocrine domains. In contrast, the regulatory interfaces through which these interactions may become operational within host physiology remain comparatively under-integrated within existing conceptual frameworks.

By reorganizing the literature through a layered structural mapping approach, the present study proposes the cellular membrane as a potentially central interface through which multigenomic interaction may become biologically operational. Membrane-associated signaling platforms appear recurrently throughout the retained literature as regulatory environments integrating host-derived signals with microbial metabolites, viral molecular inputs, and environmental biochemical factors within shared physicochemical microenvironments. Within this interpretative framework, such interfaces may function as important regulatory sites where ecological information is translated into intracellular signaling dynamics.

Within this context, physiological organization may emerge not solely from the execution of host genetic programs, but also from the continuous integration of signals originating from multiple genomic sources operating within shared biological environments. When such integrations occur repeatedly under relatively stable ecological and physiological conditions, regulatory responses may progressively converge toward coherent configurations capable of remaining energetically sustainable over time.

This systems-level interpretative perspective supports the possibility that the human organism may be understood as a symbiotic multigenomic system in which host and exogenous genomic signals participate in shaping regulatory behavior across cellular and systemic processes. Under such conditions, persistent physiological configurations may reflect stabilization dynamics emerging from distributed signal integration rather than exclusively from isolated molecular dysfunctions.

Recognizing the potential role of membrane-level signal integration within multigenomic environments therefore expands the conceptual framework through which host–microbe interaction may be interpreted. Within this perspective, human physiology may be approached not exclusively as the output of a single genome, but as the emergent behavior of interacting genomic systems operating within shared ecological and physicochemical environments. Rather than focusing exclusively on isolated pathways or individual biological agents, this approach emphasizes how regulatory influence may become organized across interacting biological systems within multiscale physiological contexts.

Future research aimed at examining how such regulatory configurations emerge, stabilize, and occasionally transition toward alternative states may provide important insights into the long-term organization of physiological dynamics in multigenomic biological systems. Clarifying how such systems-level stabilization processes reorganize under ecological and physiological perturbation may therefore represent an important step toward a more integrative understanding of human biological regulation.
